# Recent Advances in the Structural Design of Photosensitive Agent Formulations Using “Soft” Colloidal Nanocarriers

**DOI:** 10.3390/pharmaceutics12060587

**Published:** 2020-06-24

**Authors:** Agata Pucek, Beata Tokarek, Ewelina Waglewska, Urszula Bazylińska

**Affiliations:** Department of Physical and Quantum Chemistry, Faculty of Chemistry, Wroclaw University of Science and Technology, Wybrzeze Wyspianskiego 27, 50-370 Wroclaw, Poland; agata.pucek@pwr.edu.pl (A.P.); beata.tokarek@pwr.edu.pl (B.T.); ewelina.waglewska@pwr.edu.pl (E.W.)

**Keywords:** nanoemulsions, double nanoemulsions, “smart” liposomes, solid lipid nanoparticles, nanostructured lipid carriers, skin application, oral delivery, intravenous administration

## Abstract

The growing demand for effective delivery of photosensitive active compounds has resulted in the development of colloid chemistry and nanotechnology. Recently, many kinds of novel formulations with outstanding pharmaceutical potential have been investigated with an expansion in the design of a wide variety of “soft” nanostructures such as simple or multiple (double) nanoemulsions and lipid formulations. The latter can then be distinguished into vesicular, including liposomes and “smart” vesicles such as transferosomes, niosomes and ethosomes, and non-vesicular nanosystems with solid lipid nanoparticles and nanostructured lipid carriers. Encapsulation of photosensitive agents such as drugs, dyes, photosensitizers or antioxidants can be specifically formulated by the self-assembly of phospholipids or other amphiphilic compounds. They are intended to match unique pharmaceutic and cosmetic requirements and to improve their delivery to the target site via the most common, i.e., transdermal, intravenous or oral administration routes. Numerous surface modifications and functionalization of the nanostructures allow increasing their effectiveness and, consequently, may contribute to the treatment of many diseases, primarily cancer. An increasing article number is evidencing significant advances in applications of the different classes of the photosensitive agents incorporated in the ”soft” colloidal nanocarriers that deserved to be highlighted in the present review.

## 1. Introduction

There has been growing interest by many scientific groups and numerous pharmaceutical companies in the design and synthesis of novel colloidal formulations with specific features, i.e., good biocompatibility, appropriate size and structure and high colloidal stability, as well as high loading capacity and the controlled release of active compounds [1,2,3]. Many of these active agents are photosensitive, e.g., promising drugs, dyes, photosensitizers and antioxidants. Consequently, they create difficulties related to low photostability leading to ineffective delivery and poor bioavailability after transdermal, intravenous or oral administration. Taking that into consideration, it has become very important to provide high photosensitivity and sufficient protection of these delicate compounds from the external environment [4,5,6]. 

Over the last years, significant progress has been achieved in the field of novel nanocarriers engineering, involving either polymeric (e.g., polymers, polyelectrolytes and polyplexes) [7,8] or solid/liquid oleic (e.g., natural oils, waxes and phospholipids) components, as well as other additives used to stabilize the colloidal systems [5,9]. The newest research indicates that “soft” nanostructures such as nanoemulsion-origin and lipid-based formulations can significantly boost the effective delivery of photosensitive compounds. The high potential of these systems is also evidenced by the constantly growing number of papers and particularly patents, granted for their manufacture in the recent years [10,11,12,13]. 

This is due to their improved biocompatibility, enhanced permeability and better entrapment/encapsulation/loading efficiency, as well as scalability and cost effective manufacturing [14]. These advantages are extremely important in transdermal and oral applications where the surface functionalization by special ligands is not so necessary for effective delivery of the photosensitive compounds [3,15,16]. Nevertheless, in the case of intravenous administration, “soft” colloidal nanosystems obtained by self-assembly of phospholipids or other amphiphilic compounds can be easily functionalized by structural design processes [17,18]. Particularly, surface modification of emulsion or nanoemulsion systems by layering [19,20] or embedding [21,22] processes is recognized as an effective approach to incorporate the “smart” properties of the nanocarrier. Examples of the smart behavior are tumor cells targeting and modifying its distribution pattern related to the possibility to reveal the specific amount of active compounds by controlled external triggers. Layering is an approach leading to the coating of emulsion droplets stabilized by ionic surfactants with solid particles or liquid materials of the opposite charge. In this way, nanocapsules with a liquid oil core and polymer shell, i.e., core–shell nanostructures, can be obtained [19]. The embedding process involves enclosing one droplet in another liquid phase, which leads to the production of multiple (double) emulsions with micro or nanodroplet size [21]. Double emulsions are complex formulations in which both oil-in-water (O/W) and water-in-oil (W/O) dispersions exist simultaneously. Thanks to their unique double compartment structure, hybrid agents variable in their hydrophobicity may be effectively loaded into W/O/W or O/W/O phases, similarly to lipid vesicles such as liposomes, ethosomes, niosomes or transferosomes [22,23,24]. Non-vesicular lipid nanostructures, including solid lipid nanoparticles (SLNs) and nanostructured lipid carriers (NLCs), successfully tackle poor colloidal stability of nanocarriers and low solubility of the active cargo what is the main goal of all pharmaceutical and cosmetic formulations [25,26,27].

**Figure 1:**
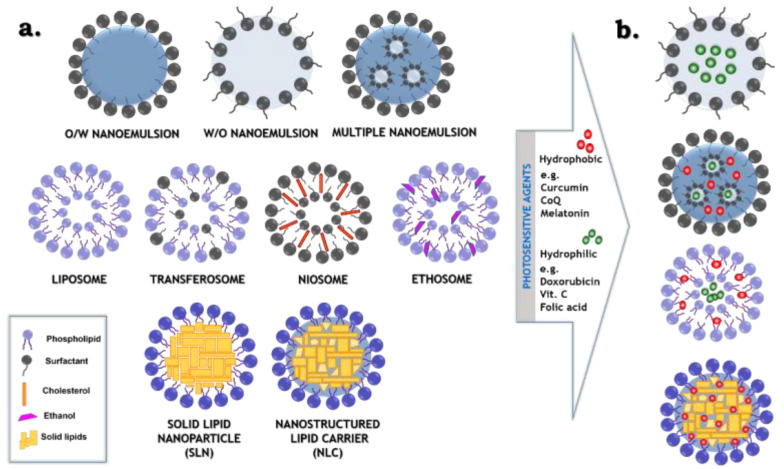
Schematic representation of most popular “soft” colloidal nanostructures (**a**) applied to solubilization/encapsulation of hydrophobic and/or hydrophilic photosensitive agents (**b**).

The present review highlights the latest advances in a variety of “soft” colloidal nanostructures (Figure 1), i.e. emulsion origin dispersions (nanoemulsions and multiple/double nanosystems) and lipid formulations, including vesicular (liposomes and modified liposomes: transferosomes, niosomes and ethosomes) as well as non-vesicular lipid nanocarriers (SLNs and NLCs). The aim of all presented formulations is to encapsulate, protect, release and therefore enhance the incorporation of photosensitive agents into the skin, stomach or tumor tissue. Special focus is put on the formulation design, its composition and engineering methods followed by recent applications in transdermal, intravenous and oral delivery of photoactive compounds. The present review was inspired by recently published paper, in which H. Zhong et al. [28] presented the most commonly used routes of pharmaceuticals administration approved by FDA, as oral, intravenous and cutaneous.

## 2. The Structure of the System

### 2.1. Nanoemulsions

Nanoemulsions are kinetically stable colloidal dispersions, also referred to as ultrafine emulsions, miniemulsions or submicron emulsions [29]. Thanks to the unique properties, such as a high surface area to volume ratio, transparent or translucent appearance, robust stability and improved biocompatibility, nanoemulsions are widely used in different areas, such as drug delivery, cosmetics, food or pharmaceuticals industries. Typically, nanoemulsion consists of water, oil and an emulsifier, which is usually the surface active agent, also called the surfactant (Figure 1). It is essential to add the emulsifier to obtain dispersed phase with nanoscale droplet size, as it has an influence on decreasing the interfacial tension, which exists between the water and the oil phase in disperse systems [30]. The size of the nanoemulsion droplets varies depending on the source and it is not strictly defined. Considering different research papers, the maximum size is determined as 100 [31], 200 [29,32] or 500 nm [33]. Moreover, many authors proposing the droplet size of nanoemulsion do not precisely provide information if they specify particle diameter or particle radius as the most recommended size of the dispersed phase. Regardless of the smaller droplet size, nanoemulsions differ from the conventional emulsion in gravitational stability (the stability increases for diameter of droplet size below 180 nm), optical properties (the solution become translucent or transparent when droplets diameters are below or about 60 nm) or improved bioavailability (better solubility of lipophilic components when the diameter of droplets is below or about 200 nm) [34]. The free energy of nanoemulsion system is very often determined by equations used in the thermodynamic description of emulsified systems. In general, all emulsions without addition of any surface active agents are thermodynamically unstable due to the fact that the interfacial area which is between the two phases, where one is dispersed in another, is much bigger in comparison to two bulk phases, which form only single interface [35]. There is a presented idea that the comparison of free energy before and after the process of emulsification gives a simplified explanation of thermodynamics of the dispersed systems. By producing small droplets, the interfacial area (Δ*A*) and configurational entropy (Δ*S*) become higher, whereas the increase of free energy related to the greater interfacial area is much bigger than the change of configurational entropy, thus it can be neglected. Finally, the equation which allows to calculate the free Gibbs energy (Δ*G*) is defined as Equation (3), whereas *γ* relates to the oil–water interfacial tension.

(1) ΔG=γΔA−TΔS

(2) |γΔA|≫|−TΔS|

(3) ΔG=γΔA

As the value of free energy of the process of emulsification is positive, the emulsion is thermodynamically unstable [36].

There has also been some confusion in scientific literature, about terms “microemulsion” and “nanoemulsion”. The prefix “nano” refers to the size in the order of 10^−9^ m, while “micro” to 10^−6^ m. According to that, microemulsions should possess bigger droplets than nanoemulsions, but in practice it is the opposite—microemulsions are characterized by smaller size of droplets than nanoemulsions. It is connected to the history of colloidal science. The term “microemulsion” was used for the first time in an article in 1961 [37], whereas the term “nanoemulsion” in 1996 [38]. As a consequence, the name “microemulsion” had already been widely used in scientific literature when the term “nanoemulsions” was introduced. The most important difference between nanoemulsions and microemulsions is their stability. Nanoemulsions, contrary to the latter, are thermodynamically unstable systems. It means that the free energy of a nanoemulsion is higher than the free energy of separate phases and, consequently, this kind of system will break down after some time, at a rate which depends on the energy barrier between separated phases and nanoemulsion [34]. This energy barrier also determines kinetic stability of the systems. To obtain a disperse system with long-term stability the energy barrier above about 20 kT is required. The kT unit relates to the potential energy of the system (k stands for Boltzmann’s constant and T for absolute temperature). In the case of Brownian motions, the energy of particles are usually less than a few kT, so an energy barrier equal or even bigger than 20 kT refers to suspension with a high stability [39]. It is due to the fact that Brownian motions are the major reason for the destabilization mechanism in nanoemulsions [40]. It is possible to improve kinetic stability by fabricating nanoemulsion with controlled microstructure (appropriate particle size distribution) or the addition of suitable stabilizers [41].

The choice of the appropriate emulsifier, usually the surfactant with amphiphilic architecture, is essential to achieve stable nanoemulsion system, which influences the manufacturing process and properties of the obtained formulation. In many cases, it is more beneficial to apply a mixture of surface active agents instead of one compound. Because of that, the composition of nanoemulsion consists usually of the water phase, the oil phase, surfactant and optionally co-surfactant [34]. There is a wide range of surface active agents, which can be applied as the emulsifiers in nanoemulsion manufacturing processes. The main classification of surfactants is based on the charge of the molecule polar group, amongst them anionic, cationic, non-ionic and zwitterionic (amphoteric) compounds are distinguished [42]. The electrical properties of emulsifiers play an important role in the formation and determining the stability of the formulation. In the case of non-ionic surfactants, the system is stabilized by repulsive steric interaction, dipoles and the presence of hydrogen bonds occurring due to interaction with the water molecules. Despite the lack of electrostatic forces, non-ionic surfactants are more commonly used than ionic ones because of their lower toxicity. They are also generally allowed for oral delivery of various substances [43]. Among the non-ionic surfactants, the most often utilized are: (i) polyoxyethylene sorbitan esters, such as Tween 80 and Tween 20; (ii) polyoxyethylene ether surfactants, such as Cremophor EL; (iii) polyglycerols; (iv) sorbitan fatty acid esters; or (v) sucrose esters [44]. To improve the stability and facilitate the formation of nanoemulsion, the combination of emulsifiers or the co-surfactant can be added to the formulation. The co-surfactant is a surface active amphiphilic molecule, that possesses too small size of the molecule to provide satisfactory stability itself, they are usually short or medium-chain alcohols [45]. Among the emulsifiers, except for surfactants, there are also other substances, which can play a role of stabilizers, such as proteins, polysaccharides and phospholipids or some polymers, such as polyvinyl alcohol [46]. 

The type of applied oil phase is very important as well. It affects the polarity, solubility, surface tension and rheology of the specific dispersion. The oil phase of nanoemulsions can be composed of various non-polar components, such as free fatty acids, monoacylglycerols, diacylglycerols, triacylglycerols, mineral oils, essential oils, flavor oils, waxes, oil-soluble vitamins or various lipophilic, often photosensitive, nutraceuticals (e.g., phytostanols, curcumin, phytosterols or carotenoids) [45]. To obtain nanoemulsion with small droplets, oils with low interfacial tension and low viscosity should be applied. This type of oil phase is disrupted in a shorter time by external forces and low oil–water interfacial tension simplifies the size reduction process due to the decreasing the amount of required energy [43]. Even though the aforementioned oils are advantageous for the process with reduction of energy demand, they are prone to destabilization processes, such as coalescence or Ostwald ripening [45]. Prevention of Ostwald ripening can be achieved by the addition of large molar volume of non-polar substances e.g., medium chain triglycerides (MCT), long-chain triglycerides (LCT) or vegetable oils, such as sesame or corn oils [47].

In recent years, there has been an increasing interest in more complex emulsions, which provide functional properties. The structural formulations are manufactured by the application of structural design methods. It is possible to distinguish three main categories of techniques applied to the process of obtaining structural nanoemulsions: layering, embedding and clustering [17]. Layering is a process based on coating droplets by the laminate, which can consist of variety of different substances, such as polymers (e.g., proteins or polysaccharides), solid particles (e.g., silica or clay), liquid droplets (oil droplets) or fibers. In this approach, droplets are stabilized by an ionic emulsifier, and, consequently, the charged particles or molecules can be deposited on them creating additional layer on their surface. Another method of the nanoemulsion structural design is the embedding process, involving enclosing one droplet in another, bigger one or another material such as different liquid phase (multiple emulsion), hydrogel matrix or solid phase (microencapsulated system). Moreover, to add additional properties to nanoemulsion, the technique defined as clustering can be applied. It is a process related to the way of aggregation state of the droplets. Controlled clustering is utilized to obtain formulation with desired viscosity or gelling properties [48].

The possibility of obtaining the colloidal system with the submicron size of the dispersed phase also depends on the production method. Those can be classified as high-energy and low-energy methods. High energy methods require external energy, which can be provided by using mechanical equipment, such as high-pressure homogenizers, rotary mixers or ultrasound generators. Whereas in the case of low-energy methods, energy essential for creating nanoemulsion comes mainly from physiochemical processes of the system [49]. 

Nanoemulsions have been widely used in the delivery of anticancer drugs, photosensitizers or therapeutic agents. In addition to the remarkable properties of nanoemulsions such as robust stability, transparent appearance and high surface area, one of the biggest advantages of these formulations is the possibility of incorporating lipophilic ingredients. The examples of that kind of compounds are photosensitive agents, such as drugs, antioxidants, photosensitizers, nutraceuticals, preservatives or flavors. Nanoemulsion also protects substances which are susceptible to oxidation or hydrolysis. Furthermore, the nanoemulsion structural design can offer prolonged action and controlled release of the loaded agents, raising the potential of the formulation to be considered as very promising carriers for the delivery of the active cargo including photosensitive compounds [30,50,51].

### 2.2. Multiple Formulations

Multiple (multiple-phase or double) emulsions are multifaceted dispersed systems with a hierarchical structure, termed also “emulsions of emulsions”. In this case, inside the continuous phase (e.g., water: W), instead of the presence of homogeneous droplets of the dispersed phase (e.g., oil: O), the droplets consisting of oil with subsequent dispersed phase (water: W) are present. It means that, in these formulations, both oil-in-water (O/W) and water-in-oil (W/O) emulsions exist simultaneously. Thus, two important types of multiple emulsions are determined: W/O/W and O/W/O. The W/O/W type (Figure 1), firstly described by Matsumoto et al. (1976), is the most commonly studied system as a potential delivery formulation in the field of food ingredients, pharmaceuticals and cosmetics [52,53]. The significant advantage of multiple emulsions is the simultaneous introduction (entrapment/encapsulation/solubilization) of two mutually immiscible (polar and non-polar) or reactive molecules onto the internal double compartment structure and improved control release of the hybrid cargo [54,55].

Multiple emulsions are highly metastable systems, as they possess two distinct types of thermodynamically unstable interfaces with opposite properties. The nature of amphiphilic molecules of surfactant adsorbed at oil–water interfaces, as well as volume, shear/agitation and temperature, plays a crucial role in the multiple emulsion formation process [56]. The most important aspect related to the Bancroft’s rule requires the application of two different surface active agents (emulsifiers) for the stabilization of W/O/W formulation: the first one, with a low hydrophilic–lipophilic balance (HLB) (from 2 to 6, i.e., Span-origin), for the W/O interface and the second one, with a high HLB (from 10 to 16, i.e., Tween or Cremophor family), for the O/W phase. A similar two-stage process is involved for the preparation of O/W/O multiple emulsions with the reverse order of surfactants of different solubility [57]. The presence of these two emulsifiers in the system, which differ in solubility, is a major source of multiple emulsions instability. The lifetime of the micrometer-size domains is significantly shortened by the rapid diffusion of the more water-soluble surfactants molecules to the droplet interface. Among other mechanisms, the formation of inverse micelles contributes to the diffusion of molecules through the oil phase [58].

The short shelf life of the double emulsions can be enhanced by reducing the micrometer droplets size down to the nanometric scale. For this reason, many approaches have been applied to modulate the emulsion double structure and stabilize the droplets size at the nanolevel, including the nanoemulsion structural design methods [55]. These strategies involve, e.g., replacing the oil phase with a lipophilic polymer/solvent phase or using biodegradable polymers from polyester family (PLGA, PLA, PGA or PCL). As a result, fabrication of very stable polymeric nanocarriers with high efficiency of hybrid cargo encapsulation such as loading DNA with the fluorescent marker, cytostatic drug with photosensitizer or other multimodal agents dedicated to theranostics purposes [21,22,23,59] is feasible. The literature also reports the examples of droplet stabilization by amphiphilic block co-polypeptides for encapsulation of pyrene and InGaP quantum dots molecules [60]. The polymerization of the inner aqueous nanodroplets by acrylamide to form polyacrylamide hydrogel has also been described [61]. 

There is also the possibility to obtain droplets with improved stability by using an appropriate preparation method, including ultrasound cavitation, high-pressure homogenization, microfluidization or ultracentrifugation. The aforementioned high-energy methods enable to fractionate droplets into a desired nanosize range. They are generally applied for the first step of W/O nanoemulsion fabrication or for both stages, to prevent the formulation from both external and internal coalescence [62]. They are also often followed by the re-emulsification of this primary W/O nanodispersion using classical low-energy emulsification in the second step. It can be done by simply replacing the oil phase with the first W/O emulsion, to finally form the W/O/W nanoemulsion with long term stability [63]. 

Double emulsions and the multiple formulations with nanodroplets, obtained by the nanoemulsion structural design methods, occur to be versatile, and useful systems. These dispersions not only meet the requirements for easy production and scale up but also, by directly using hydrophilic and lipophilic phases at the inner or external interface. They guarantee the best possible solubilization/entrapment/encapsulation/loading efficiency of both polar and non-polar molecules [55]. The second solubilization step allows for better loading and protection from degradation as well as improved release control of the active cargo from the internal phase. Thus, double nanoemulsions triggered considerable interest as “liquid reservoirs” and effective delivery systems for sensitive molecules. It relates to food (flavors, spreads, dyes and flavonoids), pharmaceutical industry (drugs, diagnostic agents and photosensitizers) and cosmetic applications (vitamins, antioxidants and anti-aging compounds). They protect the photosensitive components from external environmental reactivity such as oxidation, light and enzymes [54]. 

### 2.3. Simple and Modified “Smart” Liposomes

Currently, phospholipid vesicles, known as liposomes, are one of the most intensively explored delivery systems of active substances. The spontaneously formed vesicular carriers were first described in the 1960s by Alec Bangham [64]. Liposomes (Figure 1) are spherical structures composed of phospholipids, the main and natural components of biological membranes. These biomolecules are amphiphilic compounds containing both, hydrophobic (non-polar tails) and hydrophilic (polar head) parts. There are several types of lipids used in the liposomal formulations, for example phosphatidylcholine (PC), phosphatidylethanolamine (PE), phosphatidylglycerol (PG) or phosphatidylserine (PS). Apart from phospholipids, cholesterol, which affects the liquidity of the lipid bilayer and consequently improves the vesicle stability, can also be an important ingredient of the liposomal membrane [65]. 

The size of liposomes is one of the key parameters in designing nanoscopic carriers of active cargo. For transdermal drug delivery, it is possible to use vesicles with a diameter ≤ 300 nm, which enables the active cargo to penetrate the deeper skin layers [66]. However, in the case of liposomal systems used for delivery of the active cargo to tumor tissues, the carriers with a larger size can also be used. This is associated with the enhanced permeability and retention (EPR) effect, which is characterized by the rapid process of angiogenesis connected with the formation of new, defective and leaky capillaries. EPR effect allows nanostructures with sizes < 780 nm to penetrate pathological tissues [65]. Various sizes of liposomes can be received using different preparation methods. The selection of the appropriate method depends on many factors, among others, the final application of the phospholipid vesicles. Moreover, changes in the preparation technique, surface charge and the composition of lipid bilayer also can significantly affect the liposomes properties [67]. The most popular approach to create multilamellar vesicles (MLVs) is the thin-film hydration method preparing liposomes with the size > 500 nm. Nevertheless, this kind of vesicles is rapidly captured by the mononuclear phagocyte system (MPS) cells. To prevent the nanocarrier uptake by MPS, the size of obtained MLV dispersion can be reduced by extrusion, sonication or homogenization. Consequently, small unilamellar vesicles (SUVs) with size from 20 to 100 nm or large unilamellar vesicles (LUVs) with size >100 nm can be obtained. It is also possible to produce giant unilamellar vesicles (GUVs), which can be used to transport proteins or other macromolecules, because their size is greater than 5 µm [65,68]. 

Liposomes are very promising carriers, with high potential in the cosmetics and pharmaceutical industry. Due to their vesicular structure, they can encapsulate substances with different solubility. The hydrophilic drugs possess an affinity to the aqueous space, which is created by the phospholipid bilayer. Moreover, liposomes are non-toxic, biocompatible and biodegradable and the use of phospholipid vesicles contributes to reducing toxicity and improving the therapeutic index of the loaded cargo. Additionally, liposomes effectively protect drugs against degradation [65]. Nevertheless, the phospholipid vesicles have also some limitations, which are connected with their instability, especially in the case of oral or intravenous application. Circulation half-lives of liposomes can be increased by introducing numerous surface modifications and functionalization. This approach enables to obtain stable “smart” vesicles, which can enhance the transport of biologically active substances to target tissues. One of the example of liposome modification is surface coating using the biocompatible and hydrophilic polymer—polyethylene glycol (PEG) [69]. Such surface modified phospholipid vesicles are called “PEGylated” or “stealth” liposomes. Their steric barrier improves the efficacy of the encapsulated cargo, by preventing the nanocarrier from interactions with serum components including opsonins. As a result, the PEGylated shell protects the nanocarriers from rapid recognition and removal by the MPS [18,70]. Apart from PEG, natural (e.g., hyaluronic acid, alginate, chitosan or dextran) and synthetic (e.g., polyvinyl alcohol) polymers can also be applied to stabilize the liposomal formulations [71]. Moreover, the coating of lipid nanostructures by chitosan leads to increase in mucoadhesive properties and encapsulation efficiency. Consequently, this natural polymer is the most commonly used polycation for coating phospholipid vesicles [72]. Thanks to attaching targeting ligands (e.g., antibodies, proteins, aptamers and vitamins) to the lipid bilayer, application of modified liposomes leads to their increased accumulation at the target site, i.e. cancer cells. Besides, it is also possible to receive the theranostic liposomes, which have both diagnostic and therapeutic properties [18].

To improve the stability and flexibility of liposomes, many modifications associated with the introduction of other surfactant molecules into the vesicular structure despite of phospholipids can be applied. The results of the mentioned modifications are three generations of modern “soft” nanostructures—niosomes, transferosomes and ethosomes (Figure 1). Niosomes are the first generation of modified liposomes consisting mainly of non-ionic surfactants and cholesterol. The second generation of “smart” vesicles, composed of phospholipids and non-ionic surfactants (e.g., sodium cholate or Tween 20/60/80), is represented by transferosomes. The role of the additional surfactant, as an “edge activator”, is to destabilize the lipid bilayers. It lowers the interfacial tension, which causes the deformability of vesicles. This group is considered highly elastic and deformable [73,74]. Ethosomes are another innovative vesicular lipid formulation, i.e., the third generation that is made up of phospholipids in combination with ethanol (20–45%). The addition of alcohol increases lipid fluidity and cell membrane permeability which leads to the formation of malleable and soft vesicles. Therefore, ethanol plays an important role as an efficient permeation enhancer, which in combination with phospholipids causes a synergistic effect. This contributes to enhancing the distribution and penetration of that group of liposomes into skin [70,75]. 

Numerous modifications of the liposome structure contribute to an increase in the effectiveness and safety of pharmaceuticals delivery to the target tissue. It is also important that the high therapeutic effect of the vesicular nanocarriers is related to the reduction of the exposure of healthy tissues to the encapsulated active ingredients. Furthermore, the drug release from liposomes at the destination site is possible thanks to the action of a specific factors, such as light, temperature, pH, ultrasound or magnetic field. Consequently, the innovative nanocarriers for the delivery of biologically active substances minimalize undesired effects arising partially from the impermanence of the active cargo which can be caused by the light sensitivity [76]. 

### 2.4. Nanocarriers Based on Solid Lipids

Solid lipid-based nanocarriers have emerged in the 1990s as colloidal particles with nanometric size (between 50 and 1000 nm). They were first described by Müller’s group and divided into two generations. Solid lipid nanoparticles (SLNs) possess a core composed only of lipid or mixture of lipids, which are solid at room temperature and at the temperature of the human body. The composition consists of only solid lipids, which in the SLN matrix reveal an almost perfect, crystalline structure. The second group, nanostructured lipid carriers (NLCs), are characterized by core which, despite of the solid lipid phase also contains the addition of liquid lipid (oil). As a result, the formation of an amorphous structure (an imperfect crystal) is obtained [26,77]. Nevertheless, both types of the non-vesicular lipid nanocarriers (Figure 1) have numerous advantages. Among them the most important are: no toxicity, high biodegradation and biocompatibility, controllable size, the ability of surface functionalization and cancer targeting, high stability and loading capacity, the ability of controlled/prolonged release of encapsulated agents, increasing cellular uptake and the possibility of producing formulations in a powder form (e.g., by lyophilization/spray drying) [78,79].

The crucial components of SLNs and NLCs are solid lipids, usually present in an amount from 0.1% to 30.0% of the total formulation. Lipids used for the production of nanocarriers should meet many requirements, such as high biodegradability, good stability against decomposition (e.g., oxidation processes) and high solubility of encapsulated components [80]. Common solid lipids used in the formulation of SLN and NLC structures include triglycerides (i.e., tristearin, tripalmitin, trimyristin, rarely trilaurin and tricaprin), fatty acids (i.e., stearic, oleic, palmitic or behenic acid), monoglycerides (glyceryl monostearate, glyceryl behenate and glyceryl palmitostearate), fatty alcohols (stearyl, cetyl or lauryl alcohol) and waxes (cetyl palmitate, beeswax or carnauba wax). In NLC formulations, where the lipid matrix is supported by addition a liquid lipid (oil phase), two types of oils are mainly used: (i) saturated oils such as medium-chain triglycerides, capric/caprylic triglycerides, paraffin oil, myristyl myristate or isopropyl palmitate; and (ii) unsaturated oils such as oleic acid or various of vegetable and seed oils (from, e.g., canola, corn or soybean) [81]. To stabilize the lipid core, a surfactant or surfactants mixture is applied in an amount from 0.5% to 5.0%. There are different types of emulsifiers used in the production of SLNs and NLCs, such as non-ionic, e.g., poloxamers, pluronics or Tweens; ionic, e.g., sodium dodecyl sulfate, sodium oleate, sodium cholate, sodium deoxycholate, sodium taurodeoxycholate, cetrimonium bromide; and zwitterionic (amphoteric), e.g., phosphatidylcholine or egg/soybean lecithin, which can also be part of the lipid phase [25,81].

The non-vesicular nanocarriers based on solid lipids can be produced by using many methods with a variety of energy requirements. High energy methods include high-pressure homogenization (carried out both at low and high temperature), high-speed homogenization, emulsification–evaporation and ultrasound technique. On the other hand, processes based on solvents (i.e., solvent diffusion or solvent injection) and microemulsion/double emulsion techniques are low-energy methods of obtaining carriers [80,82]. The most common technique, characterized by simplicity and high process efficiency, is high pressure homogenization (HPH). The main advantage of this method is the possibility to scale it up, which is crucial from a technological point of view and gives a perspective for the industrial application. Additionally, HPH providing highly stable dispersions of small particles with uniform size distribution and does not require the use of organic solvents. This makes the process environmentally friendly and safe from the administration standpoint [25].

The versatility of SLNs and NLCs contributes to their widely usage as carriers of anticancer drugs, antimicrobial agents, gene vector, transdermal drugs and bioimaging agents. Due to their high biocompatibility, nanoparticles based on solid lipids can be administrated orally, topically and intravenously, through the pulmonary route or by ocular delivery. Encapsulation of photosensitive agents in SLNs or NLCs can eliminate the problems of low solubility of cargo, protect it from degradation in biological environment and thus increase the effectiveness of therapy. Selective delivery of medicines to their places of action allows improving their effectiveness and reducing the side effects. It is also worth mentioning that lipid-based nanostructures can transfer drugs by two different mechanisms: passive delivery (using the EPR effect) or active delivery mechanisms (applying surface functionalization through ligands and receptors) [26,79].

## 3. Delivery of Photosensitive Agents

### 3.1. Advances in Skin Formulations

The skin, as the largest organ of the human body, is a non-invasive and effective route of administration for many biologically active substances. The skin has many important functions; for example, it regulates temperature and prevents excessive water loss. Moreover, it protects body against external factors, i.e., ultraviolet radiation, microorganisms and toxic compounds [83,84]. The protection function is also associated with physical properties of the skin (desquamation, pH) and also the presence of many essential cells such as Langerhans (immunocompetent) cells and melanocytes (UV protection) [85].

**Figure 2:**
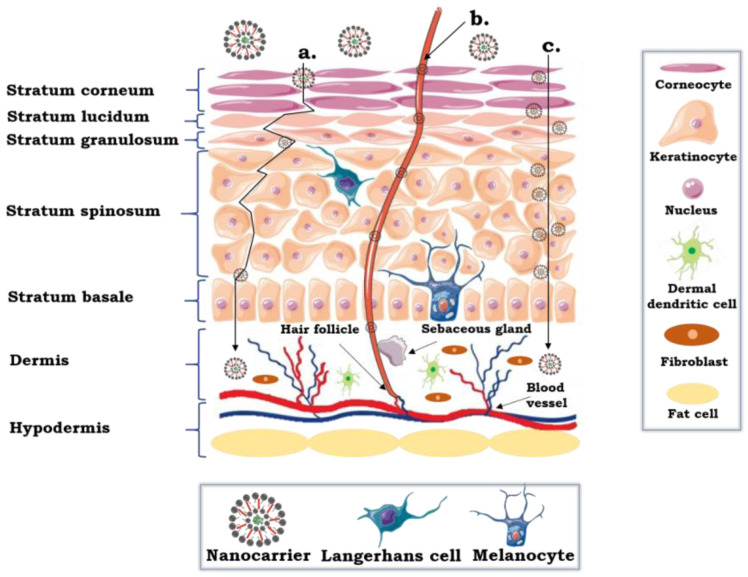
Schematic structure of the skin with possible pathways of percutaneous permeation of nanocarriers: (**a**) the intercellular; (**b**) the follicular (appendages); or (**c**) the transcellular path.

The skin has a complex construction and is made of three main layers: the epidermis, the dermis and the hypodermis (Figure 2). The outermost layer of the epidermis, the stratum corneum (SC), is the principal barrier to transdermal or topical drug delivery. This is associated with the construction of SC, composed of densely packed corneocytes. Furthermore, these cells are surrounded by the extracellular lipid matrix (the key and highly hydrophobic ingredients are ceramides, free fatty acids, cholesterol), organized as multiple lamellar bilayers. The construction is often called as “the bricks and mortar” configuration since the bricks constitute corneocytes which are embedded in the specialized lipid matrix known as the mortar [85,86,87]. 

Active agents may be transported across the epidermis by passive diffusion through three various types of pathways—the transcellular, the intercellular and the appendage routes (Figure 2) [88,89]. The intercellular route provides transport of nanoformulations by the tortuous path across the intercellular lipid matrix, between corneocyte, while the transcellular pathway allows diffusion of nanocarriers through the corneocytes. In this particular case, the nanostructures traverse alternately through hydrophilic (interior of corneocytes) and hydrophobic (extracellular lipid matrix) regions. The amount of ingredients that penetrate through the skin via intracellular route is low. The reason for that is because the active substances must partition and diffuse between these two regions [75,89]. Additionally, encapsulated cargo can penetrate through a follicular route using dermal structures, for example hair follicles with associated sebaceous glands. The additional course may be influential for the migration of nanostructures, even though hair follicles and sebaceous glands constitute around 0.1% of the skin surface [85,90]. Significantly, due to the capillary vessels proximity, the follicular route can provide accelerated transport, playing an important role when preparations are applied on the scalp due to the high density of hair follicles in this part of the body [89].

Transdermal drug delivery is an attractive and effective route for transporting active substances. It is an alternative to oral and parenteral delivery of drugs as well as to hypodermic injections. Nevertheless, a low penetration rate through the stratum corneum—the layer which limits the drug permeation is the huge problem in this route of administration. Nanostructures with specific physicochemical properties play an important role in topical drugs delivery as they can transport active cargo (e.g., photosensitive drugs) through the skin. Moreover, nanocarriers may allow penetration into deeper layers of skin, leading to prolonged cargo release [83]. There are many different types of new carrier systems dedicated to transdermal delivery, including nanoemulsions, multiple emulsions, liposomes, solid lipid nanoparticles and nanostructured lipid carriers. These nanocarriers are later formulated into creams, gels, patches or ointments which help to improve treatment efficiency in topical administration [84].

Encapsulation of photosensitive agents protects them from photodegradation which may contribute to many side effects occurring after exposure to sunlight, such as photo-allergic and phototoxic reactions. Substances are considered as photosensitive when they can absorb visible or UV radiation [123]. Many drugs with anticancer, antioxidative, antimicrobials, anti-inflammatory and anti-aging properties possess the photosensitivity. The application of photoprotective systems allows for prevention of changes of these agents as the effect of UV radiation. Therefore, nanoencapsulation leads to an increase in the therapeutic effect of drugs and other active compounds. An overview of applications for transdermal delivery using different “soft” nanoemulsion and lipid-origin nanocarriers with photosensitive cargo is presented in Table 1. 

One of the key applications provided by the transdermal route of nanoformulations loaded with photosensitive agents is the treatment of melanoma, which is one of the most aggressive skin cancers and develops from pigment producing cells called melanocytes. Melanoma cells are often resistant to immunotherapy or radiotherapy, therefore currently researchers focus on treatment based on photosensitizers and other drugs, encapsulated in carriers such as nanoemulsions or lipid-origin nanostructures [124]. Incorporation of these agents into suitable containers is also very important due to their high toxicity, low biocompatibility and poor solubility. The unique properties of photosensitizers can be used in photodynamic therapy (PDT) as a complementary treatment method. Activating them with light at the appropriate wavelength leads to the destruction of tumor cells, excluding healthy tissues [125]. Bazylińska et al. [105] developed polymer-free lipid carriers loaded with chlorin e6 or meso-tetraphenylporphine-Mn(III) chloride and showed effective internalization into Me45 and MeWo cell lines and significant cytotoxic effect after photoirradiation. Encapsulation of indocyanine green (ICG) in chitosan-coated liposomes made from 1,2-dimyristoyl-sn-glycero-3-phosphocholine and cholesterol has been successfully reported by Lee et al. [94]. The authors shown increased cellular uptake by B16-F10 melanoma cells and improved skin penetration of ICG.

The second example of a dermal disease being treated with photosensitive substances is psoriasis, characterized by red, scaly and itchy patches that appear on the skin. In the therapy of psoriasis, oral administration of drugs is inadvisable due to side effects such as hepatic toxicity. For this reason, the topical administration is necessary for safe treatment along with the increased drug local bioavailability at the diseases site [113,126]. Rajitha et al. [109] prepared an effective topical formulation of methotrexate-loaded nanoemulsion from chaulmoogra oil. They showed that the obtained formulation is characterized by enhanced skin permeation, thus it is a promising alternative for oral methotrexate administration with reduced systemic toxicity. The same drug was also co-encapsulated with etanercept by Ferreira et al. [118] in antibody-functionalized SLNs. In vitro tests showed a sustained release of methotrexate from the nanocarriers, whereas permeation studies demonstrated enhanced skin penetration of methotrexate after incorporation into SLNs. Photosensitive nanoformulations are also applied in acne treatment, a common inflammatory skin condition that can cause discomfort and visible, long-lasting scarring [127]. Examples of treatment agents for acne lesions are retinoids, such as tretinoin, efficiently encapsulated in liposomes [102] and nanoemulsions [110]. In clinical studies, the formulations result in a reduced number of acne lesions and improved skin condition with high efficacy. In addition, retinoic acid loaded into NLCs by Ghate et al. [115] demonstrated increased retention in the skin in ex vivo tests.

Researchers widely describe a preserving role of nanoformulations in hyperpigmentation treatment, a facial pigmentary disorder which causes uneven skin coloration. This illness is associated with melanin overproduction. One of the most popular whitening agents is quercetin, a natural flavonoid with antioxidant properties, which exhibits anti-tyrosinase activity. Lu et al. [112] described “nanoquercetin” applied in cosmetic formulations, which improved water solubility and skin penetration ability of quercetin thanks to the use of niosomes. Quercetin was also loaded to NLCs, stabilized by Pluronic F68 and applied as the anti-aging and UV-B protective formulation [116].

Photosensitive nanoformulations are also extensively used in accelerating wound healing. This process is very important, because it prevents infections and restores desired skin homeostasis. Chronic wounds can lead to delayed or inefficient closure. Curcumin is one of the drugs which displays the ability to heal wounds, results from its anti-infectious, antioxidant and anti-inflammatory effects. The in vivo studies and results from histopathological examination received by Ahmad et al. [106] showed that curcumin-loaded nanoemulsions are non-toxic and exhibited enhancement of wound healing effects in rats. Besides, encapsulation of curcumin in liposomes [97] provided deep penetration into the skin after in vivo tests and indicated that these nanostructures may be effective methods in enhancing the healing effect of various wounds. 

### 3.2. Advances in Intravenous Formulations 

Intravenous administration of drugs is the most common and the most effective delivery route of photosensitizers and chemotherapeutics that offers high drug availability with an insignificant delay and controlled delivery rate. Furthermore, the desired concentration of therapeutic drug can be achieved in the target tissues or cells [18,26]. The administration of compounds that are poorly absorbed through the gastrointestinal tract and drugs that cause severe pain after intramuscular or subcutaneous action can be delivered by intravenous route. Nevertheless, this type of drug administration requires experience and the presence of qualified staff due to the possible serious consequences of inappropriate application of medications [128]. 

**Figure 3:**
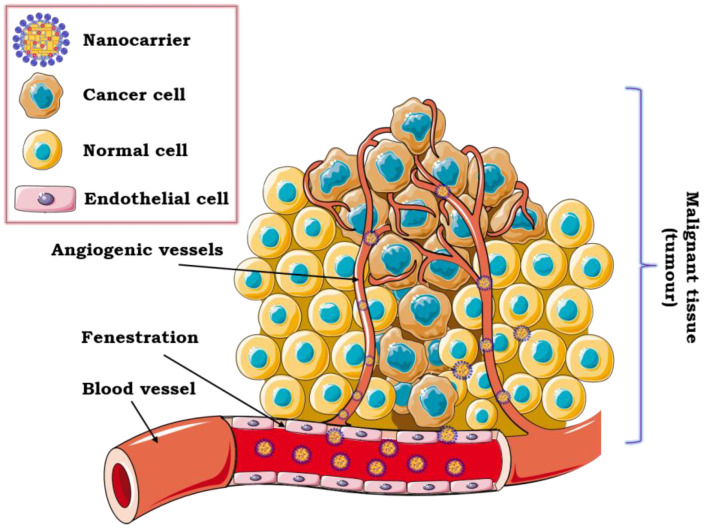
Schematic illustration of the Enhanced Permeation and Retention (EPR) effect as a mechanism of intravenous drug delivery to malignant tissue.

The efficacy of intravenous drug delivery depends on the EPR effect and the unique anatomy of the tumor, i.e., large openings between endothelial cells in malignant tissue vasculature (typically, 200–700 nm). All these aspects contribute to effective delivery of active molecules (e.g., photosensitive agents) encapsulated in the colloidal nanocarrier to the tumor site (Figure 3). 

At the same time, the usage of nanoformulations for intravenous delivery eliminates the penetration of healthy tissues due to the appropriate size of the encapsulated drug [65]. Targeting the tumor cells via blood vessels is considered to be highly effective due to its almost immediate pharmacological action. It is also a method that provides the best bioavailability, and, moreover, small doses of therapeutics can be used. However, the most serious disadvantages of this type of administration are the possibility of vein damage, tissue necrosis as well as patient discomfort and suffering [128,129]. One of the most essential aspects of obtaining safe nanoformulations for intravenous application is the sterilization process. Sterilization of water dispersions can be carried out through the action of saturated steam under pressure in an autoclave. In this case, accurate monitoring of parameters (i.e., temperature, time and pressure) is necessary to provide sufficient chemical and physical stability. Another strategy for sterilization is sterile filtration, mainly used for thermosensitive nanocarriers of small size (<220 nm). The last solution is gamma irradiation, which affects the final stability and, consequently, the safety of the formulation [130]. Table 2 summarizes the recent studies on photosensitive agents encapsulated in nanoemulsion and lipid-origin formulations dedicated to intravenous delivery of photosensitive agents.

Photosensitive compounds are widely used in photodynamic therapy (PDT), in the treatment of many diseases, mainly cancer. Photosensitizers, when exposed to a particular type of light, generate reactive oxygen species (ROS), which are highly toxic at the site of action. Selective accumulation of photosensitizers in malignant tissues, without damaging healthy cells, is possible due to their encapsulation in the appropriate nanocarrier forming the “Trojan horse” approach [125]. The use of “soft” nanostructures, as the photosensitizer drug delivery system protects also its photosensitivity against the external environment and increases local concentration improving the effectiveness of PDT. Two crucial features of photosensitizers are high photostability and substantial quantum yield leading for effective production of ROS upon irradiation. It should also have a clearly defined chemical composition and be removed from the body as soon as possible after therapy [131]. The largest group of photosensitizing agents are porphyrin compounds, for example temoporfin. It was effectively encapsulated in liposomes composed of 1,2-dipalmitoyl-sn-glycero-3-phosphatidyl-choline mixed with another lipid, without the use of a surfactant [132] and applied in PDT of ovarian cancer. A similar photosensitizer, verteporfin, was encapsulated in NLCs [133] and used to improve ovarian cancer PDT in vitro and in vivo. In turn, verteporfin-loaded multilayer oil-core nanocapsules prepared by a layer-by-layer method [134], were successfully applied in the treatment of human lung adenocarcinoma epithelial (A549) cells, cultured in vitro using multifunctional microfluidic device. Verteporfin was also co-encapsulated with cisplatin in nanocarriers obtained by double emulsion (water-in-oil-in-water) evaporation process [21]. Those formulations were applied in standard and electroporation-enhanced PDT against human ovarian (SKOV-3) cancer cells.

Another example of a porphyrin-type photosensitizer is chlorin e6. Park et al. [135] developed nanoemulsions (using soybean oil as an oil phase and PEG-*b*-PCL as an amphiphilic stabilizer), which showed effective cellular uptake and ROS generation in 4T1 mouse breast cancer cells upon laser irradiation. Chlorin e6 was also encapsulated with a cytostatic drug (paclitaxel) in NLCs [136] or with hypoxia-activated prodrug (tirapazamine) in liposomes [137] giving promising effects of synergistic combination therapy of breast cancer upon MDA-MB-231 or MCF-7 cells, respectively. Curcumin loaded into nanoemulsion [138] was capable of generating an efficient photodynamic response upon CasKi and SiHa cells, giving the potential for the treatment of cervical cancer. Furthermore, zinc-phthalocyanine type photosensitizers are used in PDT as well. Oshiro-Junior et al. [139] developed NLCs functionalized with folic acid as potential delivery systems for the alternative treatment of breast cancer. Miretti et al. [140] reported enhancement in photocytotoxicity of TAZnPc and ZnPc delivered using liposomes upon glioblastoma cells. Both types of the formulations were described as promising zinc phthalocyanine-loaded nanocarriers for use in the treatment of cancer by PDT.

Chemotherapeutic and cytostatic drugs are identified as light sensitive agents as well. Chemotherapy is a form of treatment intended to damage rapidly growing cells, usually the cancer ones. It is also used to prepare the patient for radiation therapy or before surgical removal of a tumor. Chemotherapy has been proven to prevent malignant tissue from growing; nevertheless, it can cause side effects such as peripheral neuropathy, infertility and heart or kidney problems [25]. Zheng et al. [156] synthesized SLNs from arginine-glycine-aspartic tripeptide conjugated and examined the impact of doxorubicin-loaded nanocarrier on MCF-7 cells. The results of in vitro and in vivo studies showed an improvement in chemotherapy of breast cancer cells. Li et al. [144] prepared light sensitive liposomes with special-structured phospholipid material and modified photosensitizer co-encapsulated with a cytostatic drug—doxorubicin. They described effective internalization into MCF-7 and SKOV-3 cells and combined effect of chemotherapy with PDT of the obtained system. Moreover, studies on the encapsulation of other chemotherapeutic drugs were successfully conducted. The examples are gemcitabine loaded in liposomes based on chemically modified phosphoethanolamine [143], daunorubicin in polyelectrolyte oil-core nanocarriers stabilized with novel cationic surfactants [153] and docetaxel co-encapsulated with the multifunctional peptide in SLNs [154].

Photosensitive compounds are also used in diagnostics as bioimaging agents. Darwish et al. [142] synthesized phosphatidylcholine-based liposomes co-loaded with zinc-phthalocyanine star polymer as a fluorescent marker and nitazoxanide as an anticancer drug. The results of biodistribution studies show a higher accumulation of the light-responsive formulation in the solid tumor in mice. Research on co-encapsulation of thiazole orange as a hydrophobic diagnostic dye with DNA in multiple W/O/W nanoemulsions was successfully reported by Bazylińska [22]. The biological stability studies proved the potential of the nanocarriers in in vivo gene therapy and theranostic applications. Liu et al. [145] purposed folate-targeted and oxygen/indocyanine green-loaded liposomes and examined their effective accumulation at the tumor site. IR-780-loaded nanoemulsion-based multilayer nanocapsules prepared using quaternary ammonium Gemini surfactants were also synthesized [19]. The conducted in vitro tests showed the accumulation of nanocarriers in multiple tumor cells (doxorubicin-sensitive breast, epithelial lung adenocarcinoma and skin melanoma). Pucek et al. [157] performed studies on the encapsulation of IR-780 in NLCs and imaged it in cancer cells, i.e., human epidermoid carcinoma (A431) and malignant skin melanoma (MeWo) cell lines. The same group [155] proposed co-encapsulation of IR-780 acting as a diagnostic agent and flavonoid in SLNs for bioimaging studies in human colon LoVo cells.

### 3.3. Advances in Oral Delivery

**Figure 4:**
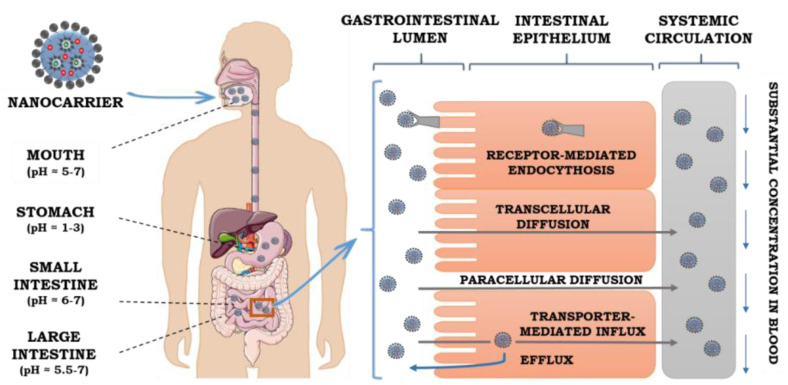
Schematic route in the digestive tract and uptake mechanisms for oral drug delivery.

Oral drug delivery is a comfortable, convenient and economical route of drug administration. It does not require special medical supplies (such as needles, syringes) or trained medical staff. Moreover, it decreases tissue damage. As a result, the oral route is considered harmless and beneficial for the patients. It can consequently contribute to the increased chance for successful treatments. Nevertheless, the pharmacological effect of drug taken orally is mainly determined by its bioavailability, which is crucial for effective delivery of the active molecules to target sites [158]. Physiological barriers, which are present in the gastrointestinal tract (Figure 4) directly impact the efficiency of oral drug delivery systems. The carrier should be able to overcome limitations connected with the characteristic physiological environment and its conditions such as variable pH, enzymes, intestinal motility or the presence of bile salts [159]. The harsh environment of the gastrointestinal area requires good stability from the drug carrier. The destructive influence of these conditions can lead to inactivation of active cargo and finally contribute to unsatisfactory therapeutics effects [160].

The dynamic process of the substance transport from the gastrointestinal lumen through the intestinal epithelium to the portal blood is called absorption. The dissolution rate, permeability and solubility are the most important factors influencing intestinal drug absorption. Solubility is a physiochemical parameter, which defines the maximum concentration of the dissolved compound in specific conditions (pH, temperature and solvent), whereas the permeability relates to the potential of active compounds to pass through the gastrointestinal membranes, which depends directly on transport mechanisms [161]. An effective drug delivery system requires the permeability and solubility balance, due to the properties of lipophilic drugs. They have a high membrane permeability and usually low aqueous solubility, so the optimization of these two factors is necessary. There is also a dependency between the degree of ionization and the value of pH. The ionized forms of active agents are better soluble in water in comparison to non-ionized ones. Thus, the ionized species are absorbed more easily in the gastrointestinal tract. The third important factor—the dissolution rate—determines the transfer of the mass to the solvent or dissolution media from the solid phase. It is a dynamic parameter that is variable over time. The dissolution rate can be a limiting factor of drug as they need to be dissolved before the absorption process [158].

From the mechanistic point of view, there are three pathways of intestinal absorption of active substances, which are schematically presented in Figure 4. Transporter-mediated absorption is controlled by receptors and transporters, which are attached to the cell membrane. They are able to carry substances in the direction of excretion (efflux) or absorption (influx). As the receptors and transporters are spaced unequally along the gastrointestinal tract, they can contribute to the side-dependent absorption process. The second absorption mechanism, passive diffusion, is based on the transport of an encapsulated cargo by the paracellular pathway. The transition of the drug occurs via the junctions between enterocytes, whereas endocytosis is a process which is characteristic for macromolecules uptake [162]. Numerous examples of nanocarriers for the oral administration of photosensitive agents are presented in Table 3. Among them, the different types of formulations such as nanoemulsions, self-nanoemulsifying drug delivery systems (SNEDDS), nanostructured lipid carriers or liposomes with various compositions (oil phase, or emulsifier) are investigated.

Curcumin is one of the compounds that is most often described as an active cargo of the nanoemulsion and lipid-origin formulations [51]. It was used for preparing the effective nanoemulsion against colon and pituitary cancer cell lines [163]. Furthermore, there is a series of formulations produced to improve the oral bioavailability of curcumin, as in the case of nanoemulsion with an extended capacity of curcumin composed of Tween 80, soybean oil and glycerol obtained by applying the emulsification inversion point method [164]. Some studies focused on the improvement of oral bioavailability of this flavonoid, applied another method such as producing a complex of that substance. The example of that approach, are curcumin–phospholipid complexes, which are based on preparing phospholipid curcumin structures and then incorporating them into SNEEDS [165]. According to those studies, the obtained formulation exhibits improved oral absorption and enhanced cytotoxic action in metastatic breast tumor cell lines. 

Additional method for improvement of curcumin bioavailability is preparing formulation consisting of curcumin and thymoquinone (another phytochemical) loaded in SNEDDS and then converting it into solid form by application of special absorbents as solid carriers [189]. The studies showed that the combined delivery system exhibited increased drug capacity and better dissolution rate which make them promising formulation for anticancer or anti-inflammatory therapies. There are also some methods for modification of the nanocarriers surface by coating them with different polymers. The example can be an application of quaternized chitosan derivatives, which allowed to obtain nanocarriers with better stability, controlled release of curcumin in simulated intestinal fluid and higher oral bioavailability [185]. Chitosan was also applied for a nanoemulsion coating, which consisted of MCT oil, Tween 80 and lecithin [174]. Structuration of that system by the layering method allows obtaining promising formulation for the delivery system of curcumin. In another investigation, conducted by Vecchione et al. [175], small emulsion droplets (110 nm) allowed obtaining the effective formulation for co-delivery of curcumin and piperine. This system has 64 times better bioavailability of curcumin in comparison to the unformulated molecules, due to different degrees of chitosan modifications. The drug was also delivered using NLCs coated by functional conjugates, e.g., *N*-acetyl-*L*-cysteine-polyethylene glycol (100)-monostearate [179]. Studies revealed the improvement of intestinal absorption of the formulation in comparison to the simple curcumin NLCs.

Another significant example of a photosensitive substance intended for oral administration is quercetin—the naturally occurring flavonoid with several relevant properties, such as anti-allergic, anti-inflammatory, anticancer and anti-viral activity [194]. According to Pangeni et al. [176], quercetin-loaded nanoemulsion, obtained by titration of aqueous phase can be a promising formulation for obesity treatment. Instead of simple nanoemulsion, multiple systems can be applied as an effective nanocarriers for the aforementioned active compound. The same group [172] obtained multiple W/O/W emulsion for efficient co-delivery of quercetin and pemetrexed (a chemotherapy drug). The formulation occurred to be promising oral delivery system, as it showed significant rise in bioavailability in comparison to a free flavonoid. Moreover, NLCs may turn out to be effective nanovehicles for quercetin as well. In the studies conducted by Sun et al. [180], the first step of obtaining effective formulation is based on the preparation of NLCs and then incorporating that in alginate hydrogel. As a result, a system with better stability, controlled release of the photosensitive agent and modified behavior in the gastrointestinal tract can be obtained. To improve the oral bioavailability of quercetin numerous other systems were produced, such as SNEDDS enhancing brain targeting [190], SNEDDS using synergistic effect of mixing antioxidants [191], modification of polymer–lipid hybrid nanoparticles by cholate [166] or lipid-based nanocarriers consisting of castor oil, lecithin and PEG 600-stearate [169].

Numerous reports also describe research on coenzyme Q10 (CoQ10) formulations [195]. CoQ10, also called ubidecarenone or ubiquinol-10, was encapsulated in nanoemulsion to decrease the oxidative damages and improve its bioavailability in the treatment of Parkinson’s disease [173]. Pharmacokinetic study exhibited almost two times better bioavailability than in CoQ10 suspension. Furthermore, Khattab et al. [188], encapsulated CoQ10 in SNEDDS, improving bioavailability of this bioactive compound and proposed the solution for treating live fibrosis and cirrhosis.

Statins are very well tolerated groups of photosensitive drugs used as lipid-lowering substances with outstanding therapeutic properties and safety profiles [196]. The literature reports a phospholipid complex system consisting of atorvastatin, incorporated in a nanoemulsion [193]. Elmowafy et al. [181] prepared other vehicles, such as the NLCs loaded with atorvastatin to improve the oral bioavailability and sustain release of that active compound. Various NLCs, including ones functionalized by polymethacrylate polymers, were also formulated for improving oral delivery of simvastatin—another antihyperlipidemic drug from the statins family [29,30].

There are also reports describing amphotericin B, an antifungal drug, with photosensitizing activity [197]. Senna et al. [167] obtained a dual strategy delivery system consisting of hydrogel and NLCs for amphotericin B. Alginate as a stimuli-sensitive polymer can be used as protection against gastric acid present in gastrointestinal track for acid-sensitive molecules. The obtained system revealed high pH sensitivity and low cytotoxicity, and it was able to control the rate of delivering by the alginate swelling ratio. IR-780 is another reported photosensitive agent which exhibits outstanding photothermal effects by absorbing NIR light. Nevertheless, this compound possesses limited photostability and poor solubility in aqueous environment. Due to that, it was effectively encapsulated in NLCs and delivered of using oral administration was possible [183].

There are also some studies revealing a novel approach for improving oral bioavailability of various other pharmaceuticals. It can be achieved by applying different vesicular nanostructures, such as niosomes used for oral delivery of antibiotics, e.g., levofloxacin [178]. There is also another way to improve oral bioavailability by using an innovative method of converting liquid into solid self-emulsifying lipid formulations employing inorganic adsorbent, as was the case of encapsulation of an antipsychotics drug, risperidone [187]. According to Teixeira et al. [177], vitamins, such as D-*α*-tocopherol, can also be efficiently loaded in nanoemulsion composed of medium-chain triglyceride, Tween 80 and Lipoid S75 to obtain the system compatible with biological fluids. Moreover, in the studies conducted by Bazylińska et al. [184], baicalein and myricetin, naturally occurring flavonoids, were effectively encapsulated in phosphatidylcholine SLNs, by using three different techniques. As a result, promising nanocarriers with improved bioavailability were obtained. There are also research works focused on improving the oral stability of carotenoids. Studies conducted by Jhan et al. [170] presented the method for efficient encapsulation of lycopene in double-loaded liposomes consisting lycopene *β*-cyclodextrin complex, cholesterol and soy lecithin, whereas simple liposomes were found to be effective nanocarriers for *β*-carotene [171].

## 4. Concluding Remarks and Future Perspectives

Recent advances in the application of “soft” colloids with a wide range of internal structures have opened broad perspectives on the structural design of photosensitive agent formulations. Among them, nanoemulsion and lipid-origin carriers have become the most promising photoprotective nanocarriers, since they possess no toxicity, high biodegradability and biocompatibility properties as well as controllable size and increasing cellular uptake. They may facilitate efficient delivery of different photosensitive active cargo such as photosensitizers, chemotherapeutics, flavonoids and vitamins, using various routes of administration, i.e., transdermal, oral and intravenous. Nevertheless, some key issues need to be improved for the successful application of the “soft” nanocarriers in clinical trials. Based on the extensive literature study, it is proved that both the composition and preparation methods of nanoemulsions, multiple formulations, liposomes, SLNs and NLCs are the key aspects of their final applications. Special attention should be paid to the use of non-toxic, natural and bioinspired components including phospholipids, biosurfactants and natural polymers. Consequently, the future prospective studies should be focused on preparing the nanoformulations stabilized by non-ionic surfactants with natural lipid phase possessing high encapsulation efficiency (>80%) and the particle size of 100–150 nm. The effort should be also put to enhance the colloidal stability of nanocarriers and the sustain control release of photosensitive cargo, used mainly for intravenous administration, by applying structural design techniques such as layering, embedding or clustering approach. It is possible by single- or multi-layered functionalization with different natural biopolymers such as proteins or polysaccharides. The example of that kind of modification is coating nanocarriers by “smart” targeting ligands, e.g., hyaluronic or folic acid moieties. Furthermore, the “smart” nanoformulations are characterized by increased efficiency of the active cargo delivery, reduced risk of systemic toxicity and improved bioavailability due to the sensitivity to external triggers such as temperature, pH, ionic strength, enzyme or oxidative/reductive surroundings. Increased penetration of nanostructures, loaded with the photosensitive active agents, leads to effective treatment for many diseases such as melanoma, psoriasis, vitiligo, acne, hyperpigmentation, Parkinson’s disease and plays an important role in PDT, UV protection, antioxidant activity and diagnostics. Moreover, the unique properties of nanocarriers including the different polarity of encapsulated photosensitive compounds provide novel and promising functionality making them promising structures for various applications in pharmacy, materials science or nanomedicine.

## Figures and Tables

**Table 1:** Selected photosensitive agents encapsulated by nanoemulsion and lipid-origin formulations and their applications in transdermal delivery.

Formulation	Surfactant/Co-Surfactant (Non-Lipid Origin)	Oil/Lipid	Photosensitive Cargo	D_H_ [nm]	EE [%]	Cargo Detection Method	Application	Ref
Ethosomes	-	Phosphatidylcholine	5-Aminolevulinic acid	81	53	FM	Hypertrophic scars	[91]
Ethosomes	Polyethyleneimine, sodium cholate	Phosphatidylcholine, cholesterol	Curcumin, doxorubicin	50–350	-	FM	Melanoma	[92]
Ethosomes	Cremophor A25	Phosphatidylcholine	Ferrous chlorophyllin	383	78	UV-Vis spectroscopy, CLSM	Squamous cell carcinoma of the skin, PDT	[93]
Chitosan-coated liposomes	-	Phosphatidylcholine		201	68			
Chitosan-coated liposomes	-	DMPC, cholesterol	Indocyanine green	231–1983	-	UV-Vis spectroscopy, FM	Melanoma, PDT	[94]
Lipid nanocapsules	Solutol HS 15, Cremophor EL, Labrafac Lipophile WL 1349	Lipoid S75-3	Quercetin	27–54	90–96	HPLC with ultraviolet detector	Anti-inflammatory activity, psoriasis, atopic dermatitis	[95,96]
Liposomes	Cremophor EL	DPPC	Quercetin	179	68	HPLC with ultraviolet detector	UV-protection	
Liposomes	-	Phosphatidylcholine, cholesterol	Curcumin	189–395	76–84	UV–Vis spectroscopy, FM	Wound healing	[97]
Liposomes	Oleic acid	Phosphatidylcholine	Folic acid	120–280	6–70	CLSM	Treatment of micronutrient deficiencies	[98]
Liposomes	-	Phosphatidylcholine, cholesterol, DOTAP, DSPG	Ascorbic acid	161–190	17–58	HPLC with diode array detector, FM	Anti-aging, UV-A protection	[99]
Liposomes	-	DPPC	Curcumin	104–133	31–43	HPLC with ultraviolet detector	Anti-inflammatory activity, psoriasis, melanoma	[100]
Liposomes	-	HSPC, cholesterol	Doxorubicin, celecoxib	121–142	98–99	UV-Vis spectroscopy	Melanoma	[101]
Liposomes	Dicetyl phosphate	Phosphatidylcholine, cholesterol	Tretinoin	318–485	38–73	UV-Vis spectroscopy	Acne	[102]
Peptide functionalized liposomes	Sodium cholate	Phosphatidylcholine, cholesterol	Vemurafenib	73–105	98	UV-Vis spectroscopy	Melanoma	[103]
Aspasomes	Dicetyl phosphate	Cholesterol, ascorbyl palmitate	Melatonin	287–950	52–91	UV-Vis spectroscopy	Androgenic alopecia	[104]
Cubosomes	Propylene glycol	Glycerol monooleate, Lipoid S75	Chlorin e6	138	97	UV-Vis spectroscopy, CLSM	Melanoma, PDT	[105]
			TPP-Mn	146	91			
Nanoemulsions	Tween 80/ PEG 400	Clove oil	Curcumin	93	-	DSC	Wound healing, anti-inflammatory activity	[106]
Nanoemulsions	Rapeseed lecithin	Rapeseed oil	Coenzyme Q10	123–158	93	UV-Vis spectroscopy, epi-FM	Anti-wrinkle and anti-inflammatory activities, UV-protection, skin disease (e.g., facial vitiligo)	[107]
Nanoemulsions	Tween 80/ Transcutol HP	Isopropyl myristate	Coenzyme Q10	11	-	HPLC	Anti-wrinkle activity	[108]
Nanoemulsions	Tween 80/ ethanol	Chaulmoogra oil	Methotrexate	34	88	UV-Vis spectroscopy, FM	Psoriasis	[109]
Nanoemulsions	Tween 80	Crodamol GTCC	Tretinoin	116	99	UV-Vis spectroscopy	Acne	[110]
Nanoemulsions	Pluronic F68	Clove oil	8-Methoxypsoralen	91	-	HPLC with ultraviolet detector	Psoriasis, vitiligo	[111]
	Cremophor RH40	Sweet fennel oil		68	-			
Niosomes	Span 60, Cremophor RH40	-	Quercetin	97	87	HPLC	Hyperpigmentation	[112]
Niosomes	Span 60	Cholesterol	Acitretin	369	90	UV-Vis spectroscopy	Psoriasis	[113]
NLCs	Tween 80, Labrafil M 2130 CS	Capryol 90, Captex 355, Geleol, Apifil	Folic acid	50–94	95–98	UV-Vis spectroscopy	Anti-aging	[114]
NLCs	Tween 80, Span 60	Avacado oil, grape seed oil/stearic acid, cetyl alcohol, glyceryl monostearate	All-trans retinoic acid	67	>98	HPLC with ultraviolet detector	Acne, photo-aging, eczema, psoriasis	[115]
NLCs	Pluronic F68	Calendula oil, Illipe butter	Quercetin	130	97	UV-Vis spectroscopy	Anti-aging, UV-B protection	[116]
SLNs	Poloxamer 188	Dynasan 114, soy lecithin	Ropinirole	211	77	HPLC	Parkinson’s disease	[117]
NLCs		Dynasan 114, Capryol 90, soy lecithin		193	84			
Antibody functionalized SLNs	Tween 80	Cetyl palmitate	Methotrexate	292–356	85–88	HPLC with photodiode array detector	Psoriasis	[118]
SLNs	Tween 80	Precirol ATO 5	Curcumin	51	93	HPLC	Hyperpigmentation irritant contact dermatitis	[119]
Transferosomes	Tween 20, Tween 80, Span 20, Cremophor A25	Phosphatidylcholine	Ferrous chlorophyllin	284–651	37–56	UV-Vis spectroscopy	Melanoma	[120]
Transferosomes	Tween 80	Phosphatidylcholine	Retinyl palmitate	300	100	HPLC with photodiode array detector, FM	Anti-aging, hyperpigmentation	[121]
Transferosomes	Tween 80	Phosphatidylcholine	All-trans retinoic acid	48–87	99	HPLC, CLSM	Wound healing, treatment of deep partial-thickness burns	[122]

**Abbreviations:** CLSM, confocal laser scanning microscopy; D_H_, hydrodynamic diameter; DMPC, 1,2-dimyristoyl-sn-glycero-3-phosphocholine; DOTAP, 1,2-dioleoyl-3-trimethylammoniopropane; DPPC, 1,2-dipalmitoyl-sn-glycero-3-phosphocholine; DSC, differential scanning calorimetry; DSPG, 1,2-distearoyl-sn-glycero-3-phospho-(1′-rac-glycerol); EE, entrapment efficiency; FM, fluorescence microscopy; HPLC, high performance liquid chromatography; HSPC, hydrogenated soya bean phosphatidylcholine; NLC, nanostructured lipid carrier; PDT, photodynamic therapy; SLN, solid lipid nanoparticle; TPP-Mn, meso-tetraphenylporphine-Mn(III) chloride.

**Table 2:** Selected photosensitive agents encapsulated by nanoemulsion and lipid-origin formulations and their applications in intravenous delivery.

Formulation	Surfactant/Co-Surfactant (Non-Lipid Origin)	Oil/Lipid	Photosensitive Cargo	D_H_ [nm]	EE [%]	Cargo Detection Method	Application	Ref
Liposomes	-	DPPC/cholesterol, DPPC/DPPE-mPEG_5000_ or DPPC/TEL	Temoporfin	106–129	78–90	UV-Vis spectroscopy, CLSM	PDT	[132]
Magnetic photosensitive liposomes	DDAB	DSPC, cholesterol	Indocyanine green	222	12	UV-Vis spectroscopy	PTT/PDT	[141]
Liposomes	Triton X-100	Phosphatidylcholine	Zinc-phthalocyanine star polymer, nitazoxanide	87	-	UV-Vis spectroscopy	Bioimaging, PDT	[142]
Liposomes	-	DPPC, cholesterol, DOPE, CHEMS	Gemcitabine, pheophorbide A	102	37	HPLC, CLSM	PDT, chemotherapy	[143]
Antibody functionalized liposomes	-	Phosphatidylcholine, cholesterol, DSPE-PEG_2000_	Doxorubicin, modified indocyanine green	128	>90	UV-Vis spectroscopy, FS	PDT, chemotherapy	[144]
Liposomes	-	DPPC, DPPG, DSPE-PEG_2000_-folate, cholesterol	Indocyanine green	301	96	UV-Vis spectroscopy, FS, CLSM	Diagnostics, PTT, sono-PDT photo-sonodynamic combined therapy	[145]
Liposomes	-	DPPC, cholesterol	ZnPc, TAZnPc	102–190	74–75	UV-Vis spectroscopy, FS	PDT	[140]
Liposomes	-	Lipoid S100	Temoporfin	141	82	HPLC with fluorescence detector	PDT	[146]
Liposomes	-	Phosphatidylcholine cholesterol	IR-780	130	67	UV-Vis spectroscopy, CLSM	PDT	[147]
Liposomes	-	Lecithin, DSPE-PEG_2000_, cholesterol	Chlorin e6	162	82	UV-Vis spectroscopy, FS, CLSM	Diagnostics, PDT	[137]
Nanoemulsions	Anionic dicephalic surfactants C_n_H_2n+1_-*N*-(CH_2_COONa)_2_ *n* = 10, 12 or 14	Isopropyl myristate or palm oil	Verteporfin or *meso*-tetraphenylporphyrin	129–170	85–98	UV-Vis spectroscopy	PDT	[148]
Nanoemulsions	Poloxamer 188	Lipoid S100	Curcumin	199	-	UV-Vis spectroscopy, FM, FS	PDT	[138]
Nanoemulsions	Pluronic F127	Clove oil	ZnPc	30–202	-	UV-Vis spectroscopy	PDT	[149]
Nanoemulsions	Pluronic F68	Miglyol 812 N, Epikuron TM	AlClPc	133	>99	HPLC	Diagnosis, PDT	[150]
Nanoemulsions	PEG-*b*-PCL	Soybean oil	Chlorin e6	220	-	FM, FS	PDT	[135]
Multiple nanoemulsions (polymeric double-core NCs)	Di-C_12_DMAB and Cremophor A25, Cremophor RH 40 or Poloxamer 407	PEG-PLGA, PEG-PCL, PEG-PDLLA in DCM	DNA, thiazole orange	143–184	72–95	UV-Vis spectroscopy	Gene therapy, bioimaging	[22]
Multiple nanoemulsions (polymeric double-core NCs)	Span 80, Cremophor A25	PLGA in DCM	NaYF_4_:Er^3+^,Yb^3+^NPs	134–265	-	NIR spectroscopy and spectrofluorimetry	NIR-induced imaging	[151]
Multiple nanoemulsions (”smart” double-core polymeric NCs)	Di-C_12_DMAB, Cremophor A25	PLGA, PEG-PLGA, FA-PLGA in DCM	Verteporfin, cisplatin	187–200	88–97	UV-Vis spectroscopy, CLSM, FACS	Combined chemo- and EP-PDT	[21]
Multiple nanoemulsions (”smart” double-core polymeric NCs)	Span 80, Rosulfan A	PLGA in DCM	NaYF_4_:Er^3+^,Yb^3+^NPs, Rose Bengal	127–154	-	NIR spectroscopy and spectrofluorimetry	Theranostics, NIR-induced imaging and PDT	[23]
Multiple nanoemulsions (double-core polymeric NCs)	Span 80, Cremophor A25	PLGA in DCM	NaYF_4_:Er^3+^,Yb^3+^NPs+, Rose Bengal	150–158	-	NIR spectroscopy and spectrofluorimetry CLSM	Theranostics, NIR-induced bioimaging and PDT	[152]
Nanoemulsion-based multilayer NCs	Quaternary ammonium gemini surfactants: d(DDA)PBr and d(DDA)BBr	Isopropyl myristate, oleic acid	IR-780	101–119	>90	UV-Vis spectroscopy, FM, CLSM	NIR-induced bioimaging	[19]
Nanoemulsion-based multilayer NCs	Cationic surfactant C_12_(TAPAMS)_2_	Oleic acid	Daunorubicin	103–120	86–96	UV-Vis spectroscopy, CLSM	Chemotherapy	[153]
Nanoemulsion-based multilayer NCs	Cationic surfactant C_12_(TAPAMS)_2_	Oleic acid	Verteporfin	118	92	UV-Vis spectroscopy, FM, CLSM	Diagnostics, PDT	[134]
SLNs	Tween 80	Glyceryl monostearate, stearic acid, soya lecithin	Docetaxel	79–111	87–90	HPLC, FM	Chemotherapy	[154]
SLNs	Tween 80	Cetyl palmitate, Phospholipon 90G	IR-780	134–237	22–63	UV-Vis spectroscopy, CLSM	EP-PDT	[155]
SLNs	Myrj 52	Glycerol monostearate, lecithin	Doxorubicin	81–96	89–90	HPLC, FM	Chemotherapy	[156]
NLCs	Myrj S40	Suppocire NB, soybean oil, Lipoid S75	Verteporfin	50	>95	HPLC, FM, CLSM	PDT	[133]
NLCs	Pluronic F127, Polyoxyethylene 40 stearate, ethoxylated hydrogenated castor oil	Capric/caprylic acid triglycerides	Zinc phthalocyanine	165	63	FS	PDT	[139]
NLCs	Tween 80	Cetyl palmitate, Miglyol 812 N, (CLA)PC	IR-780	159–228	-	CLSM	Bioimaging	[157]
NLCs	Cremophor RH40, DSPE-PEG_2000_	Precirol ATO5, and Maisine 35-1	Chlorin e6 and paclitaxel	121	93–94	HPLC, FM, CLSM	PDT, chemotherapy	[136]

**Abbreviations:** C_12_(TAPAMS)_2_, *N*,*N*-bis[3,30-(trimethylammonio)propyl]dodecanamide dimethylsulfate; CHEMS, cholesteryl hemisuccinate; (CLA)PC, 1,2-di(conjugated)linoleoyl-sn-glycero-3-phosphocholine; CLSM, confocal laser scanning microscopy; d(DDA)BBr, *N*,*N*,*N*′,*N*′-tetramethyl-*N*,*N*′-di(dodecyl)-butylenediammonium; d(DDA)PBr, *N*,*N*,*N*′,*N*′-tetramethyl-*N*,*N*′-di(dodecyl)-ethylenediammonium bromide; DDAB, dimethyldioctadecyl ammonium bromide; D_H_, hydrodynamic diameter; DOPE, 1,2-dioleoyl-sn-glycero-3-phosphoethanolamine; DPPC, 1,2-dipalmitoyl-sn-glycero-3-phosphatidylcholine; DPPE-mPEG_5000_, 1,2-dipalmitoyl-sn-glycero-3-phosphoethanolamine-*N*-[methoxy(polyethylene glycol)-5000]; DPPG, 1,2-dipalmitoyl-sn-glycero-3-phospho-(1′-rac-glycerol) (sodium salt); DSPC, 1,2-distearoyl-sn-glycero-3-phosphocholine; DSPE-mPEG_2000_, 1,2-distearoyl-sn-glycero-3-phosphoethanolamine-*N*-[methoxy(polyethylene-glycol)-2000]; DSPE-PEG_2000_, 1,2-distearoyl-sn-glycero-3-phosphoethanolamine-*N*-[amino(poly(ethylene glycol))2000]; DSPE-PEG_2000_-folate, 1,2-distearoyl-sn-glycero-3-phosphoethanolamine-*N*-[folate(polyethylene glycol)-2000]; EE, entrapment efficiency; EP-PDT, electroporation-supported photodynamic therapy; FACS, fluorescence activated cell sorting; FM, fluorescence microscopy; FS, fluorescence spectroscopy; HPLC, high performance liquid chromatography; NC, nanocarrier; NLC, nanostructured lipid carrier; PDT, photodynamic therapy; PTT, photothermal therapy; SLN, solid lipid nanoparticle; TEL, tetraether lipids.

**Table 3:** Selected photosensitive agents encapsulated by nanoemulsion and lipid-origin formulations and their applications in oral delivery.

Formulation	Surfactant /Co-Surfactant (Non- Lipid Origin)	Oil/Lipid	Photosensitive Cargo	D_H_ [nm]	EE [%]	Cargo Detection Method	Application	Ref
Cholate-modified polymer-lipid hybrid nanoparticles	-	PLGA, Lipoid S100	Quercetin	110	96	HPLC, CLSM	Antileukemic activity	[166]
Dual alginate-lipid nanocarriers	Tween 20, Span 80, Kolliphor P188	Glyceryl monostearate, Miglyol 812N	Amphotericin B	83–120	78–81	HPLC with photodiode array detector	Antimicrobial/ antifungal-PDT	[167]
Gel-like lipid-based drug delivery systems	PEG 400 caprylic/capric glycerides/PEG-15 hydroxystearate	PEG 300 oleic glycerides, propylene glycol monolaurate and monocaprylate	Simvastatin	13–23	-	UV-Vis spectroscopy	Antihyperlipidemic activity	[168]
Lipid-based nanocarriers	PEG 660-stearate	Castor oil, Phospholipon 80	Quercetin	20	-	HPLC with ultraviolet detector, NMR spectroscopy	Antioxidant and anti-inflammatory activity	[169]
Double-loaded liposomes	-	Phosphatidylcholine, cholesterol	Lycopene	143–652	58–84	UV−Vis spectroscopy, DSC, FTIR	Cardioprotective activity	[170]
Liposomes	-	Phosphatidylcholine, cholesterol	*β*-carotene	129	86	UV−Vis spectroscopy	Functional foods and supplements	[171]
Multiple nanoemulsions	Labrasol/Tween 80, Cremophor EL, PEG 400	Labrafil M 1944 CS	Quercetin	15	-	CLSM	Anticancer therapy	[172]
Nanoemulsions	Tween 20, Tween 80, Solutol HS 15, Unitop FFT 40/Transcutol P,ethanol, PEG 400	Vitamin E, Capmul MCM, Labrafac Lipophile WL 1349, Captex, Capryol 90	Coenzyme Q10	20–31	-	HPLC with ultraviolet detector	Parkinson’s disease treatment	[173]
Nanoemulsions	Tween 80	Soybean oil	Curcumin	198–272	-	UV–Vis spectroscopy	Food formulations	[164]
Nanoemulsions	Tween 80	MCT oil, lecithin	Curcumin	114	95	UV–Vis spectroscopy	Functional food and beverage system	[174]
Nanoemulsions	-	Soybean oil, Lipoid E80	Curcumin	110	-	CLSM, STED	Anti-inflammatory and anticancer activities	[175]
Nanoemulsions	Tween 80/TPGS	Kollisolv MCT 70	Curcumin	17	98	UV–Vis spectroscopy	Anticancer activity against pituitary and colon cell lines	[163]
Nanoemulsions	Tween 20, Tween 80, Span 80, Labrasol/ Cremophor EL, PEG 400, Transcutol HP, Plurol Oleique CC	Castor oil, oleic acid, Capryol 90, Labrafil M 1944	Quercetin	19–126	-	HPLC with ultraviolet detector	Obesity treatment	[176]
Nanoemulsions	Tween 80	Lipoid S75, Miglyol 812 N	D-*α*-tocopherol	65–90	-	-	Antioxidant activity	[177]
Niosomes	Non-ionic surfactant BRD-BG	Cholesterol	Levofloxacin	190	68	HPLC, FTIR	Antibiotic therapy	[178]
NLCs	-	Phosphatidylcholine, cholesterol oleate,	Curcumin	141	92	HPLC, CLSM	Antioxidant and anticancer activity	[179]
NLCs	Tween 80	Glycerol monostearate, octyl and decyl glycerate	Quercetin	86	98	UV–Vis spectroscopy	Anti-inflammatory and anticancer activities	[180]
NLCs	Pluronic F68, Tween 80	Gelucire 43/01, Capryol PGMC, GMS, lecithin	Atorvastatin	163–866	76–97	UV-Vis spectroscopy	Antihyperlipidemic activity	[181]
NLCs	Pluronic F68	Stearic acid, oleic acid, lecithin	Simvastatin	169	40–76	UV-Vis spectroscopy, FTIR	Antihyperlipidemic activity	[182]
NLCs	-	Labrafac CC, trilaurin, soy lecithin	IR-780	170	-	UV−Vis spectroscopy	Photothermal anticancer therapies	[183]
SLNs	-	Phosphatidylcholine, Polawax NF	Baicalein, myricetin, flavonoids cocrystals	45–104	51–92	UV–Vis spectroscopy, FTIR, XRPD	Antioxidant, antitumor and anti-inflammatory activities	[184]
SLNs	TPGS	Cholesterol, palmitic acid	Curcumin	139	93	HPLC with ultraviolet detector, FTIR	Brain gliomas and Alzheimer’s disease treatments	[185]
SLNs	Poloxamer 188	Dynasan 112, egg lecithin	Rosuvastatin calcium	67	94	HPLC	Antihyperlipidemic activity	[186]
SELFs	HCO-30, TO-106V, Transcutol P	Coconut oil, CremerCOOR, MCT 70/30, Capmul MCM, Imwitor 988, 308	Risperidone	16–111	-	FTIR	Antipsychotic activity	[187]
SNEDDS	Cremophor EL, Labrasol, Tween 80/Transcutol	Isopropyl myristate	Coenzyme Q10	11–13	-	HPLC	Protection against liver fibrosis and cirrhosis	[188]
SNEDDS	Tween 80, PEG 400	Castor oil	Curcumin	83	-	HPLC, UV-Vis spectroscopy	Anticancer activity against metastatic breast cancer cells	[165]
SNEDDS	Transcutol P, Cremophor EL, Cremophor RH40, hydrogenated castor oil	Black seed oil, Imwitor 988	Curcumin	18–25	-	FTIR, ultra-HPLC	Anti-inflammatory and anticancer treatments	[189]
SNEDDS	PEG 400, Tween 20, Tween 60, Tween 80, Labrasol, Cremophor EL	Sesame oil, oleic acid, isopropyl myristate, olive oil, ethyl oleate	Quercetin	27–249	-	UV–Vis spectroscopy, ultra-HPLC	Brain tumor	[190]
SNEDDS	Cremophor RH40, Labrafil1944 CS	Capmul MCM EP	Quercetin, resveratrol	62–214	-	HPLC, CLSM	Antioxidant therapies	[191]
SNEDDS	Lauroglycol FCC, Tween 80/Transcutol P	Castor oil	Fisetin	154–157	58–105	HPLC with photodiode array detector	The cancer, Neurodegenerative disorders treatments	[192]
Nanoemulsions	Pluronic F68, Tween 80, sodium oleate/glycerin	Soybean oil, ovolecithin	Atorvastatin	123–151	98	HPLC, FTIR	Antihyperlipidemic activity	[193]

**Abbreviations:** CLSM, confocal laser scanning microscopy; D_H_, hydrodynamic diameter; DSC, differential scanning calorimetry; EE, entrapment efficiency; FTIR, Fourier transform infrared spectroscopy; HPLC, high performance liquid chromatography; NLC, nanostructured lipid carrier; NMR, nuclear magnetic resonance; PDT, photodynamic therapy; PLGA, poly(lactic-*co*-glycolic acid); SELFs, solid self-emulsifying lipid formulations; SLN, solid lipid nanoparticle; SNEDDS, self-nanoemulsifying drug delivery systems; STED, stimulated emission depletion microscopy; TPGS, D-*α*-tocopherol polyethylene glycol 1000 succinate; XRPD, X-ray powder diffraction.

## References

[1] Avramović N., Mandić B., Savić-Radojević A., Simić T. (2020). Polymeric Nanocarriers of Drug Delivery Systems in Cancer Therapy. Pharmaceutics.

[2] Siepmann J., Faham A., Clas S.D., Boyd B.J., Jannin V., Bernkop-Schnürch A., Zhao H., Lecommandoux S., Evans J.C., Allen C. (2019). Lipids and polymers in pharmaceutical technology: Lifelong companions. Int. J. Pharm..

[3] Hsu C.Y., Wang P.W., Alalaiwe A., Lin Z.C., Fang J.Y. (2019). Use of lipid nanocarriers to improve oral delivery of vitamins. Nutrients.

[4] Li T., Yan L. (2018). Functional polymer nanocarriers for photodynamic therapy. Pharmaceuticals.

[5] Chen B.H., Inbaraj B.S. (2019). Nanoemulsion and nanoliposome based strategies for improving anthocyanin stability and bioavailability. Nutrients.

[6] Ali A., Ahmad U., Akhtar J., Badruddeen, Khan M.M. (2019). Engineered nano scale formulation strategies to augment efficiency of nutraceuticals. J. Funct. Foods.

[7] Venditti I. (2019). Morphologies and functionalities of polymeric nanocarriers as chemical tools for drug delivery: A review. J. King Saud Univ. Sci..

[8] Bordat A., Boissenot T., Nicolas J., Tsapis N. (2019). Thermoresponsive polymer nanocarriers for biomedical applications. Adv. Drug Deliv. Rev..

[9] Zeb A., Arif S.T., Malik M., Shah F.A., Din F.U., Qureshi O.S., Lee E.S., Lee G.Y., Kim J.K. (2019). Potential of nanoparticulate carriers for improved drug delivery via skin. J. Pharm. Investig..

[10] Paliwal R., Paliwal S.R., Kenwat R., Kurmi B.D., Sahu M.K. (2020). Solid lipid nanoparticles: A review on recent perspectives and patents. Expert Opin. Ther. Pat..

[11] Sanchez-Barcelo E., Mediavilla M. (2014). Recent Patents on Light Based Therapies: Photodynamic Therapy, Photothermal Therapy and Photoimmunotherapy. Recent Pat. Endocr. Metab. Immune Drug Discov..

[12] El-Hammadi M.M., Arias J.L. (2019). An update on liposomes in drug delivery: A patent review (2014-2018). Expert Opin. Ther. Pat..

[13] Patel R.B., Thakore S.D., Patel M.R. (2016). Recent Survey on Patents of Nanoemulsions. Curr. Drug Deliv..

[14] Date T., Nimbalkar V., Kamat J., Mittal A., Mahato R.I., Chitkara D. (2018). Lipid-polymer hybrid nanocarriers for delivering cancer therapeutics. J. Control. Release.

[15] Maqsoudlou A., Assadpour E., Mohebodini H., Jafari S.M. (2020). Improving the efficiency of natural antioxidant compounds via different nanocarriers. Adv. Colloid Interface Sci..

[16] Md S., Haque S., Madheswaran T., Zeeshan F., Meka V.S., Radhakrishnan A.K., Kesharwani P. (2017). Lipid based nanocarriers system for topical delivery of photosensitizers. Drug Discov. Today.

[17] McClements D.J. (2012). Advances in fabrication of emulsions with enhanced functionality using structural design principles. Curr. Opin. Colloid Interface Sci..

[18] Riaz M.K., Riaz M.A., Zhang X., Lin C., Wong K.H., Chen X., Zhang G., Lu A., Yang Z. (2018). Surface functionalization and targeting strategies of liposomes in solid tumor therapy: A review. Int. J. Mol. Sci..

[19] Bazylińska U., Saczko J. (2016). Nanoemulsion-templated polylelectrolyte multifunctional nanocapsules for DNA entrapment and bioimaging. Colloids Surf. B Biointerfaces.

[20] Bazylinska U., Saczko J., Zielinska K., Wilk K.A. (2012). Novel multilayer IR-786-Loaded nanocarriers for intracellular delivering: Characterization, imaging, and internalization in human cancer cell lines. Chem. Lett..

[21] Bazylińska U., Kulbacka J., Chodaczek G. (2019). Nanoemulsion structural design in co-encapsulation of hybrid multifunctional agents: Influence of the smart PLGA polymers on the nanosystem-enhanced delivery and electro-photodynamic treatment. Pharmaceutics.

[22] Bazylińska U. (2017). Rationally designed double emulsion process for co-encapsulation of hybrid cargo in stealth nanocarriers. Colloids Surf. A Physicochem. Eng. Asp..

[23] Wawrzyńczyk D., Cichy B., Zarȩba J.K., Bazylińska U. (2019). On the interaction between up-converting NaYF4:Er3^+^,Yb3^+^ nanoparticles and Rose Bengal molecules constrained within the double core of multifunctional nanocarriers. J. Mater. Chem. C.

[24] Grimaldi N., Andrade F., Segovia N., Ferrer-Tasies L., Sala S., Veciana J., Ventosa N. (2016). Lipid-based nanovesicles for nanomedicine. Chem. Soc. Rev..

[25] Haider M., Abdin S.M., Kamal L., Orive G. (2020). Nanostructured Lipid Carriers for Delivery of Chemotherapeutics: A Review. Pharmaceutics.

[26] Bayón-Cordero L., Alkorta I., Arana L. (2019). Application of solid lipid nanoparticles to improve the efficiency of anticancer drugs. Nanomaterials.

[27] Mathew A., Marotta F., Kumar D.S., Alexander M., Vaiserman (2017). Nanotechnology in Anti-Aging: Nutraceutical Delivery and Related Applications. Anti-Aging Drugs: From Basic Research to Clinical Practice.

[28] Zhong H., Chan G., Hu Y., Hu H., Ouyang D. (2018). A comprehensive map of FDA-approved pharmaceutical products. Pharmaceutics.

[29] Solans C., Izquierdo P., Nolla J., Azemar N., Garcia-Celma M.J. (2005). Nano-emulsions. Curr. Opin. Colloid Interface Sci..

[30] Gupta A., Eral H.B., Hatton T.A., Doyle P.S. (2016). Nanoemulsions: Formation, properties and applications. Soft Matter.

[31] Rao J., McClements D.J. (2012). Lemon oil solubilization in mixed surfactant solutions: Rationalizing microemulsion & nanoemulsion formation. Food Hydrocoll..

[32] Huang Q., Yu H., Ru Q. (2010). Bioavailability and delivery of nutraceuticals using nanotechnology. J. Food Sci..

[33] Anton N., Benoit J.P., Saulnier P. (2008). Design and production of nanoparticles formulated from nano-emulsion templates-A review. J. Control. Release.

[34] McClements D.J. (2012). Nanoemulsions versus microemulsions: Terminology, differences, and similarities. Soft Matter.

[35] Lopetinsky R.J.G., Masliyah J.H., Xu Z. (2006). Solids-Stabilized Emulsions: A Review. Colloidal Particles at Liquid Interfaces.

[36] Clements D.J.M.C. (2005). Food Emulsions: Principles, Practices and Techniques.

[37] Schulman J.H., Montagne J.B. (1961). Formation of Microemulsions By Amino Alkyl Alcohols. Ann. N. Y. Acad. Sci..

[38] Calvo P., Vila-Jato J.L., Alonso M.J. (1996). Comparative in vitro evaluation of several colloidal systems, nanoparticles, nanocapsules, and nanoemulsions, as ocular drug carriers. J. Pharm. Sci..

[39] Elimelech M., Gregory J., Jia X., Williams R.A. (1995). Surface Interaction Potentials. Particle Deposition & Aggregation.

[40] Erramreddy V., Ghosh S. (2016). Gelation in nanoemulsion: Structure formation and rheological behavior. Emulsions.

[41] Bhattacharjee K. (2019). Importance of Surface Energy in Nanoemulsion. Nanoemulsions - Prop. Fabr. Appl..

[42] Nakama Y. (2017). Surfactants. Cosmetic Science and Technology: Theoretical Principles and Applications.

[43] Pavoni L., Perinelli D.R., Bonacucina G., Cespi M., Palmieri G.F. (2020). An overview of micro-and nanoemulsions as vehicles for essential oils: Formulation, preparation and stability. Nanomaterials.

[44] Flanagan J., Singh H. (2006). Microemulsions: A potential delivery system for bioactives in food. Crit. Rev. Food Sci. Nutr..

[45] McClements D.J., Rao J. (2011). Food-Grade nanoemulsions: Formulation, fabrication, properties, performance, Biological fate, and Potential Toxicity. Crit. Rev. Food Sci. Nutr..

[46] Aswathanarayan J.B., Vittal R.R. (2019). Nanoemulsions and Their Potential Applications in Food Industry. Front. Sustain. Food Syst..

[47] Pavoni L., Pavela R., Cespi M., Bonacucina G., Maggi F., Zeni V., Canale A., Lucchi A., Bruschi F., Benelli G. (2019). Green micro-and nanoemulsions for managing parasites, vectors and pests. Nanomaterials.

[48] Paximada P., Mandala I., Assadpour E., Mehrnia M.A. (2017). 2 – Encapsulation by nanoemulsions. Nanoencapsulation Technologies for the Food and Nutraceutical Industries.

[49] McClements D.J. (2011). Edible nanoemulsions: Fabrication, properties, and functional performance. Soft Matter.

[50] Jaiswal M., Dudhe R., Sharma P.K. (2015). Nanoemulsion: An advanced mode of drug delivery system. 3 Biotech.

[51] Calmon M., Bonfim C., Monteleoni L., Candido N., Quintana S., Melli P., Primo F., Adamantino C., Antonio T., Rahal P. (2018). Effect of curcumin-nanoemulsion associated with photodynamic therapy in HPV-16 E6 positive vulvar carcinoma cell lines. Clin. Cancer Res..

[52] Matsumoto S., Kita Y., Yonezawa D. (1976). An attempt at preparing water-in-oil-in-water multiple-phase emulsions. J. Colloid Interface Sci..

[53] Garti N., Bisperink C. (1998). Double emulsions: Progress and applications. Curr. Opin. Colloid Interface Sci..

[54] Giri T.K., Choudhary C., Ajazuddin, Alexander A., Badwaik H., Tripathi D.K. (2013). Prospects of pharmaceuticals and biopharmaceuticals loaded microparticles prepared by double emulsion technique for controlled delivery. Saudi Pharm. J..

[55] Sheth T., Seshadri S., Prileszky T., Helgeson M.E. (2020). Multiple nanoemulsions. Nat. Rev. Mater..

[56] Leister N., Karbstein H.P. (2020). Evaluating the Stability of Double Emulsions—A Review of the Measurement Techniques for the Systematic Investigation of Instability Mechanisms. Colloids and Interfaces.

[57] Schmidts T., Dobler D., Guldan A.C., Paulus N., Runkel F. (2010). Multiple W/O/W emulsions-Using the required HLB for emulsifier evaluation. Colloids Surf. A Physicochem. Eng. Asp..

[58] Lokhande S.S., Namita N., Phalke N.N., Raje V.N., More S.S. (2018). An Update Review on Recent Advancements in Multiple Emulsion. Int. J. Res. Sci. Innov..

[59] Gharieh A., Khoee S., Mahdavian A.R. (2019). Emulsion and miniemulsion techniques in preparation of polymer nanoparticles with versatile characteristics. Adv. Colloid Interface Sci..

[60] Hanson J.A., Chang C.B., Graves S.M., Li Z., Mason T.G., Deming T.J. (2008). Nanoscale double emulsions stabilized by single-component block copolypeptides. Nature.

[61] Ding S., Anton N., Akram S., Er-Rafik M., Anton H., Klymchenko A., Yu W., Vandamme T.F., Serra C.A. (2017). A new method for the formulation of double nanoemulsions. Soft Matter.

[62] Clegg P.S., Tavacoli J.W., Wilde P.J. (2016). One-step production of multiple emulsions: Microfluidic, polymer-stabilized and particle-stabilized approaches. Soft Matter.

[63] Sigward E., Mignet N., Rat P., Dutot M., Muhamed S., Guigner J.M., Scherman D., Brossard D., Crauste-Manciet S. (2013). Formulation and cytotoxicity evaluation of new self-emulsifying multiple W/O/W nanoemulsions. Int. J. Nanomed..

[64] Bozzuto G., Molinari A. (2015). Liposomes as nanomedical devices. Int. J. Nanomed..

[65] Olusanya T.O.B., Ahmad R.R.H., Ibegbu D.M., Smith J.R., Elkordy A.A. (2018). Liposomal drug delivery systems and anticancer drugs. Molecules.

[66] Danaei M., Dehghankhold M., Ataei S., Hasanzadeh Davarani F., Javanmard R., Dokhani A., Khorasani S., Mozafari M.R. (2018). Impact of particle size and polydispersity index on the clinical applications of lipidic nanocarrier systems. Pharmaceutics.

[67] Bulbake U., Doppalapudi S., Kommineni N., Khan W. (2017). Liposomal formulations in clinical use: An updated review. Pharmaceutics.

[68] Ortega V., Giorgio S., De Paula E. (2017). Liposomal formulations in the pharmacological treatment of leishmaniasis: A review. J. Liposome Res..

[69] Sercombe L., Veerati T., Moheimani F., Wu S.Y., Sood A.K., Hua S. (2015). Advances and challenges of liposome assisted drug delivery. Front. Pharmacol..

[70] Zylberberg C., Matosevic S. (2016). Pharmaceutical liposomal drug delivery: A review of new delivery systems and a look at the regulatory landscape. Drug Deliv..

[71] Lombardo D., Calandra P., Barreca D., Magazù S., Kiselev M.A. (2016). Soft interaction in liposome nanocarriers for therapeutic drug delivery. Nanomaterials.

[72] Barba A.A., Bochicchio S., Bertoncin P., Lamberti G., Dalmoro A. (2019). Coating of nanolipid structures by a novel simil-microfluidic technique: Experimental and theoretical approaches. Coatings.

[73] Hua S. (2015). Lipid-based nano-delivery systems for skin delivery of drugs and bioactives. Front. Pharmacol..

[74] Benson H.A.E. (2017). Elastic liposomes for topical and transdermal drug delivery. Methods Mol. Biol..

[75] Van Tran V., Moon J.Y., Lee Y.C. (2019). Liposomes for delivery of antioxidants in cosmeceuticals: Challenges and development strategies. J. Control. Release.

[76] Wang Y., Kohane D.S. (2017). External triggering and triggered targeting strategies for drug delivery. Nat. Rev. Mater..

[77] Li Q., Cai T., Huang Y., Xia X., Cole S.P.C., Cai Y. (2017). A review of the structure, preparation, and application of NLCs, PNPs, and PLNs. Nanomaterials.

[78] Tapeinos C., Battaglini M., Ciofani G. (2017). Advances in the design of solid lipid nanoparticles and nanostructured lipid carriers for targeting brain diseases. J. Control. Release.

[79] Mishra V., Bansal K.K., Verma A., Yadav N., Thakur S., Sudhakar K., Rosenholm J.M. (2018). Solid lipid nanoparticles: Emerging colloidal nano drug delivery systems. Pharmaceutics.

[80] Katouzian I., Faridi Esfanjani A., Jafari S.M., Akhavan S. (2017). Formulation and application of a new generation of lipid nano-carriers for the food bioactive ingredients. Trends Food Sci. Technol..

[81] Gordillo-Galeano A., Mora-Huertas C.E. (2018). Solid lipid nanoparticles and nanostructured lipid carriers: A review emphasizing on particle structure and drug release. Eur. J. Pharm. Biopharm..

[82] Ganesan P., Narayanasamy D. (2017). Lipid nanoparticles: Different preparation techniques, characterization, hurdles, and strategies for the production of solid lipid nanoparticles and nanostructured lipid carriers for oral drug delivery. Sustain. Chem. Pharm..

[83] Palmer B.C., DeLouise L.A. (2016). Nanoparticle-enabled transdermal drug delivery systems for enhanced dose control and tissue targeting. Molecules.

[84] Nagula R.L., Wairkar S. (2019). Recent advances in topical delivery of flavonoids: A review. J. Control. Release.

[85] Sala M., Diab R., Elaissari A., Fessi H. (2018). Lipid nanocarriers as skin drug delivery systems: Properties, mechanisms of skin interactions and medical applications. Int. J. Pharm..

[86] Jijie R., Barras A., Boukherroub R., Szunerits S. (2017). Nanomaterials for transdermal drug delivery: Beyond the state of the art of liposomal structures. J. Mater. Chem. B.

[87] Khezri K., Saeedi M., Maleki Dizaj S. (2018). Application of nanoparticles in percutaneous delivery of active ingredients in cosmetic preparations. Biomed. Pharmacother..

[88] Shaker D.S., Ishak R.A.H., Ghoneim A., Elhuoni M.A. (2019). Nanoemulsion: A review on mechanisms for the transdermal delivery of hydrophobic and hydrophilic drugs. Sci. Pharm..

[89] Carter P., Narasimhan B., Wang Q. (2019). Biocompatible nanoparticles and vesicular systems in transdermal drug delivery for various skin diseases. Int. J. Pharm..

[90] Nastiti C.M.R.R., Ponto T., Abd E., Grice J.E., Benson H.A.E., Roberts M.S. (2017). Topical nano and microemulsions for skin delivery. Pharmaceutics.

[91] Zhang Z., Liu Y., Chen Y., Li L., Lan P., He D., Song J., Zhang Y. (2019). Transdermal Delivery of 5-Aminolevulinic Acid by Nanoethosome Gels for Photodynamic Therapy of Hypertrophic Scars. ACS Appl. Mater. Interfaces.

[92] Ma L., Wang X., Wu J., Zhang D., Zhang L., Song X., Hong H., He C., Mo X., Wu S. (2019). Polyethylenimine and sodium cholate-modified ethosomes complex as multidrug carriers for the treatment of melanoma through transdermal delivery. Nanomedicine.

[93] Nasr S., Rady M., Gomaa I., Syrovet T., Simmet T., Fayad W., Abdel-Kader M. (2019). Ethosomes and lipid-coated chitosan nanocarriers for skin delivery of a chlorophyll derivative: A potential treatment of squamous cell carcinoma by photodynamic therapy. Int. J. Pharm..

[94] Lee E.H., Lim S.J., Lee M.K. (2019). Chitosan-coated liposomes to stabilize and enhance transdermal delivery of indocyanine green for photodynamic therapy of melanoma. Carbohydr. Polym..

[95] Hatahet T., Morille M., Hommoss A., Devoisselle J.M., Müller R.H., Bégu S. (2018). Liposomes, lipid nanocapsules and smartCrystals^®^: A comparative study for an effective quercetin delivery to the skin. Int. J. Pharm..

[96] Hatahet T., Morille M., Shamseddin A., Aubert-Pouëssel A., Devoisselle J.M., Bégu S. (2017). Dermal quercetin lipid nanocapsules: Influence of the formulation on antioxidant activity and cellular protection against hydrogen peroxide. Int. J. Pharm..

[97] Choudhary V., Shivakumar H., Ojha H. (2019). Curcumin-loaded liposomes for wound healing: Preparation, optimization, in-vivo skin permeation and bioevaluation. J. Drug Deliv. Sci. Technol..

[98] Kapoor M.S., D’Souza A., Aibani N., Nair S.S., Sandbhor P., Kumari D., Banerjee R. (2018). Stable Liposome in Cosmetic Platforms for Transdermal Folic acid delivery for fortification and treatment of micronutrient deficiencies. Sci. Rep..

[99] Maione-Silva L., De Castro E.G., Nascimento T.L., Cintra E.R., Moreira L.C., Cintra B.A.S., Valadares M.C., Lima E.M. (2019). Ascorbic acid encapsulated into negatively charged liposomes exhibits increased skin permeation, retention and enhances collagen synthesis by fibroblasts. Sci. Rep..

[100] Campani V., Scotti L., Silvestri T., Biondi M., De Rosa G. (2020). Skin permeation and thermodynamic features of curcumin-loaded liposomes. J. Mater. Sci. Mater. Med..

[101] Ahmed K.S., Shan X., Mao J., Qiu L., Chen J. (2019). Derma roller^®^ microneedles-mediated transdermal delivery of doxorubicin and celecoxib co-loaded liposomes for enhancing the anticancer effect. Mater. Sci. Eng. C.

[102] Rahman S.A., Abdelmalak N.S., Badawi A., Elbayoumy T., Sabry N., El Ramly A. (2016). Tretinoin-loaded liposomal formulations: From lab to comparative clinical study in acne patients. Drug Deliv..

[103] Zou L., Ding W., Zhang Y., Cheng S., Li F., Ruan R., Wei P., Qiu B. (2018). Peptide-modified vemurafenib-loaded liposomes for targeted inhibition of melanoma via the skin. Biomaterials.

[104] Hatem S., Nasr M., Moftah N.H., Ragai M.H., Geneidi A.S., Elkheshen S.A. (2018). Melatonin vitamin C-based nanovesicles for treatment of androgenic alopecia: Design, characterization and clinical appraisal. Eur. J. Pharm. Sci..

[105] Bazylińska U., Kulbacka J., Schmidt J., Talmon Y., Murgia S. (2018). Polymer-free cubosomes for simultaneous bioimaging and photodynamic action of photosensitizers in melanoma skin cancer cells. J. Colloid Interface Sci..

[106] Ahmad N., Ahmad R., Al-Qudaihi A., Alaseel S.E., Fita I.Z., Khalid M.S., Pottoo F.H. (2019). Preparation of a novel curcumin nanoemulsion by ultrasonication and its comparative effects in wound healing and the treatment of inflammation. RSC Adv..

[107] Kaci M., Belhaffef A., Meziane S., Dostert G., Menu P., Velot, Desobry S., Arab-Tehrany E. (2018). Nanoemulsions and topical creams for the safe and effective delivery of lipophilic antioxidant coenzyme Q10. Colloids Surf. B Biointerfaces.

[108] El-Leithy E.S., Makky A.M., Khattab A.M., Hussein D.G. (2018). Optimization of nutraceutical coenzyme Q10 nanoemulsion with improved skin permeability and anti-wrinkle efficiency. Drug Dev. Ind. Pharm..

[109] Rajitha P., Shammika P., Aiswarya S., Gopikrishnan A., Jayakumar R., Sabitha M. (2019). Chaulmoogra oil based methotrexate loaded topical nanoemulsion for the treatment of psoriasis. J. Drug Deliv. Sci. Technol..

[110] Sabouri M., Samadi A., Ahmad Nasrollahi S., Farboud E.S., Mirrahimi B., Hassanzadeh H., Nassiri Kashani M., Dinarvand R., Firooz A. (2018). Tretinoin loaded nanoemulsion for acne vulgaris: Fabrication, physicochemical and clinical efficacy assessments. Skin Pharmacol. Physiol..

[111] Barradas T.N., Senna J.P., Cardoso S.A., Nicoli S., Padula C., Santi P., Rossi F., De Holanda e Silva K.G., Mansur C.R.E. (2017). Hydrogel-thickened nanoemulsions based on essential oils for topical delivery of psoralen: Permeation and stability studies. Eur. J. Pharm. Biopharm..

[112] Lu B., Huang Y., Chen Z., Ye J., Xu H., Chen W., Long X. (2019). Niosomal nanocarriers for enhanced skin delivery of quercetin with functions of anti-tyrosinase and antioxidant. Molecules.

[113] Hashim I.I.A., El-Magd N.F.A., El-Sheakh A.R., Hamed M.F., El-Gawad A.E.G.H.A. (2018). Pivotal role of acitretin nanovesicular gel for effective treatment of psoriasis: Ex vivo–in vivo evaluation study. Int. J. Nanomedicine.

[114] Ammar H.O., Ghorab M.M., Mostafa D.M., Ibrahim E.S. (2016). Folic acid loaded lipid nanocarriers with promoted skin antiaging and antioxidant efficacy. J. Drug Deliv. Sci. Technol..

[115] Ghate V.M., Kodoth A.K., Raja S., Vishalakshi B., Lewis S.A. (2019). Development of MART for the Rapid Production of Nanostructured Lipid Carriers Loaded with All-Trans Retinoic Acid for Dermal Delivery. AAPS PharmSciTech.

[116] Pivetta T.P., Silva L.B., Kawakami C.M., Araújo M.M., Del Lama M.P.F.M., Naal R.M.Z.G., Maria-Engler S.S., Gaspar L.R., Marcato P.D. (2019). Topical formulation of quercetin encapsulated in natural lipid nanocarriers: Evaluation of biological properties and phototoxic effect. J. Drug Deliv. Sci. Technol..

[117] Dudhipala N., Gorre T. (2020). Neuroprotective Effect of Ropinirole Lipid Nanoparticles Enriched Hydrogel for Parkinson’s Disease: In Vitro, Ex Vivo, Pharmacokinetic and Pharmacodynamic Evaluation. Pharmaceutics.

[118] Ferreira M., Barreiros L., Segundo M.A., Torres T., Selores M., Costa Lima S.A., Reis S. (2017). Topical co-delivery of methotrexate and etanercept using lipid nanoparticles: A targeted approach for psoriasis management. Colloids Surf. B Biointerfaces.

[119] Shrotriya S., Ranpise N., Satpute P., Vidhate B. (2018). Skin targeting of curcumin solid lipid nanoparticles-engrossed topical gel for the treatment of pigmentation and irritant contact dermatitis. Artif. Cells, Nanomed. Biotechnol..

[120] Rady M., Gomaa I., Afifi N., Abdel-Kader M. (2018). Dermal delivery of Fe-chlorophyllin via ultradeformable nanovesicles for photodynamic therapy in melanoma animal model. Int. J. Pharm..

[121] Pena-Rodríguez E., Moreno M.C., Blanco-Fernandez B., González J., Fernández-Campos F. (2020). Epidermal delivery of retinyl palmitate loaded transfersomes: Penetration and biodistribution studies. Pharmaceutics.

[122] Lu K.J., Wang W., Xu X.L., Jin F.Y., Qi J., Wang X.J., Kang X.Q., Zhu M.L., Huang Q.L., Yu C.H. (2019). A dual deformable liposomal ointment functionalized with retinoic acid and epidermal growth factor for enhanced burn wound healing therapy. Biomater. Sci..

[123] Blakely K.M., Drucker A.M., Rosen C.F. (2019). Drug-Induced Photosensitivity—An Update: Culprit Drugs, Prevention and Management. Drug Saf..

[124] Beiu C., Giurcaneanu C., Grumezescu A.M., Holban A.M., Popa L.G., Mihai M.M. (2020). Nanosystems for Improved Targeted Therapies in Melanoma. J. Clin. Med..

[125] Abrahamse H., Hamblin M.R. (2016). New photosensitizers for photodynamic therapy. Biochem. J..

[126] Rendon A., Schäkel K. (2019). Psoriasis pathogenesis and treatment. Int. J. Mol. Sci..

[127] Babaie S., Del Bakhshayesh A.R., Ha J.W., Hamishehkar H., Kim K.H. (2020). Invasome: A novel nanocarrier for transdermal drug delivery. Nanomaterials.

[128] Chenthamara D., Subramaniam S., Ramakrishnan S.G., Krishnaswamy S., Essa M.M., Lin F.H., Qoronfleh M.W. (2019). Therapeutic efficacy of nanoparticles and routes of administration. Biomater. Res..

[129] Youn Y.S., Bae Y.H. (2018). Perspectives on the past, present, and future of cancer nanomedicine. Adv. Drug Deliv. Rev..

[130] Wacker M. (2013). Nanocarriers for intravenous injection - The long hard road to the market. Int. J. Pharm..

[131] Kwiatkowski S., Knap B., Przystupski D., Saczko J., Kędzierska E., Knap-Czop K., Kotlińska J., Michel O., Kotowski K., Kulbacka J. (2018). Photodynamic therapy – mechanisms, photosensitizers and combinations. Biomed. Pharm..

[132] Ali S., Amin M.U., Ali M.Y., Tariq I., Pinnapireddy S.R., Duse L., Goergen N., Wölk C., Hause G., Jedelská J. (2020). Wavelength dependent photo-cytotoxicity to ovarian carcinoma cells using temoporfin loaded tetraether liposomes as efficient drug delivery system. Eur. J. Pharm. Biopharm..

[133] Michy T., Massias T., Bernard C., Vanwonterghem L., Henry M., Guidetti M., Royal G., Coll J.L., Texier I., Josserand V. (2019). Verteporfin-loaded lipid nanoparticles improve ovarian cancer photodynamic therapy in vitro and in vivo. Cancers.

[134] Tokarska K., Bułka M., Bazylińska U., Jastrzębska E., Chudy M., Dybko A., Wilk K.A., Brzózka Z. (2016). Evaluation of nanoencapsulated verteporfin’s cytotoxicity using a microfluidic system. J. Pharm. Biomed. Anal..

[135] Park C., Yoo J., Lee D., Jang S.Y., Kwon S., Koo H. (2019). Chlorin e6-loaded PEG-PCL nanoemulsion for photodynamic therapy and in vivo drug delivery. Int. J. Mol. Sci..

[136] Zhang Q., Zhao J., Hu H., Yan Y., Hu X., Zhou K., Xiao S., Zhang Y., Feng N. (2019). Construction and in vitro and in vivo evaluation of folic acid-modified nanostructured lipid carriers loaded with paclitaxel and chlorin e6. Int. J. Pharm..

[137] Zhang K., Zhang Y., Meng X., Lu H., Chang H., Dong H., Zhang X. (2018). Light-triggered theranostic liposomes for tumor diagnosis and combined photodynamic and hypoxia-activated prodrug therapy. Biomaterials.

[138] De Matos R.P.A., Calmon M.F., Amantino C.F., Villa L.L., Primo F.L., Tedesco A.C., Rahal P. (2018). Effect of Curcumin-Nanoemulsion Associated with Photodynamic Therapy in Cervical Carcinoma Cell Lines. Biomed Res. Int..

[139] Oshiro-Junior J.A., Sato M.R., Boni F.I., Santos K.L.M., De Oliveira K.T., De Freitas L.M., Fontana C.R., Nicholas D., McHale A., Callan J.F. (2020). Phthalocyanine-loaded nanostructured lipid carriers functionalized with folic acid for photodynamic therapy. Mater. Sci. Eng. C.

[140] Miretti M., Tempesti T.C., Prucca C.G., Baumgartner M.T. (2020). Zn phthalocyanines loaded into liposomes: Characterization and enhanced performance of photodynamic activity on glioblastoma cells. Bioorganic Med. Chem..

[141] Anilkumar T.S., Lu Y.J., Chen H.A., Hsu H.L., Jose G., Chen J.P. (2019). Dual targeted magnetic photosensitive liposomes for photothermal/photodynamic tumor therapy. J. Magn. Magn. Mater..

[142] Darwish W.M., Bayoumi N.A., El-Kolaly M.T. (2018). Laser-responsive liposome for selective tumor targeting of nitazoxanide nanoparticles. Eur. J. Pharm. Sci..

[143] Kim D.H., Im B.N., Hwang H.S., Na K. (2018). Gemcitabine-loaded DSPE-PEG-PheoA liposome as a photomediated immune modulator for cholangiocarcinoma treatment. Biomaterials.

[144] Li Q., Li W., Di H., Luo L., Zhu C., Yang J., Yin X., Yin H., Gao J., Du Y. (2018). A photosensitive liposome with NIR light triggered doxorubicin release as a combined photodynamic-chemo therapy system. J. Control. Release.

[145] Liu Y., Chen S., Sun J., Zhu S., Chen C., Xie W., Zheng J., Zhu Y., Xiao L., Hao L. (2019). Folate-Targeted and Oxygen/Indocyanine Green-Loaded Lipid Nanoparticles for Dual-Mode Imaging and Photo-sonodynamic/Photothermal Therapy of Ovarian Cancer in Vitro and in Vivo. Mol. Pharm..

[146] Wallenwein C.M., Nova M.V., Janas C., Jablonka L., Gao G.F., Thurn M., Albrecht V., Wiehe A., Wacker M.G. (2019). A dialysis-based in vitro drug release assay to study dynamics of the drug-protein transfer of temoporfin liposomes. Eur. J. Pharm. Biopharm..

[147] Yang Y., Yang X., Li H., Li C., Ding H., Zhang M., Guo Y., Sun M. (2019). Near-infrared light triggered liposomes combining photodynamic and chemotherapy for synergistic breast tumor therapy. Colloids Surf. B Biointerfaces.

[148] Bazylińska U., Frąckowiak R., Brzózka Z., Wilk K.A. (2017). The effect of anionic dicephalic surfactants on fabrication of varied-core nanocarriers for sustained release of porphyrin photosensitizers. J. Photochem. Photobiol. B Biol..

[149] De Oliveira De Siqueira L.B., Da Silva Cardoso V., Rodrigues I.A., Vazquez-Villa A.L., Dos Santos E.P., Da Costa Leal Ribeiro Guimarães B., Dos Santos Cerqueira Coutinho C., Vermelho A.B., Junior E.R. (2017). Development and evaluation of zinc phthalocyanine nanoemulsions for use in photodynamic therapy for *Leishmania* spp.. Nanotechnology.

[150] Oliveira L.T., De Paula M.A., Roatt B.M., Garcia G.M., Silva L.S.B., Reis A.B., De Paula C.S., Vilela J.M.C., Andrade M.S., Pound-Lana G. (2017). Impact of dose and surface features on plasmatic and liver concentrations of biodegradable polymeric nanocapsules. Eur. J. Pharm. Sci..

[151] Bazylińska U., Wawrzyńczyk D. (2017). Encapsulation of TOPO stabilized NaYF4:Er3^+^, Yb3^+^ nanoparticles in biocompatible nanocarriers: Synthesis, optical properties and colloidal stability. Colloids Surf. A Physicochem. Eng. Asp..

[152] Bazylińska U., Wawrzyńczyk D., Szewczyk A., Kulbacka J. (2020). Engineering and biological assessment of double core nanoplatform for co-delivery of hybrid fluorophores to human melanoma. J. Inorg. Biochem..

[153] Bazylińska U., Pietkiewicz J., Rossowska J., Chodaczek G., Gamian A., Wilk K.A. (2017). Polyelectrolyte Oil-Core Nanocarriers for Localized and Sustained Delivery of Daunorubicin to Colon Carcinoma MC38 Cells: The Case of Polysaccharide Multilayer Film in Relation to PEG-ylated Shell. Macromol. Biosci..

[154] Kadari A., Pooja D., Gora R.H., Gudem S., Kolapalli V.R.M., Kulhari H., Sistla R. (2018). Design of multifunctional peptide collaborated and docetaxel loaded lipid nanoparticles for antiglioma therapy. Eur. J. Pharm. Biopharm..

[155] Kulbacka J., Pucek A., Kotulska M., Dubińska-Magiera M., Rossowska J., Rols M.P., Wilk K.A. (2016). Electroporation and lipid nanoparticles with cyanine IR-780 and flavonoids as efficient vectors to enhanced drug delivery in colon cancer. Bioelectrochemistry.

[156] Zheng G., Zheng M., Yang B., Fu H., Li Y. (2019). Improving breast cancer therapy using doxorubicin loaded solid lipid nanoparticles: Synthesis of a novel arginine-glycine-aspartic tripeptide conjugated, pH sensitive lipid and evaluation of the nanomedicine in vitro and in vivo. Biomed. Pharm..

[157] Pucek A., Niezgoda N., Kulbacka J., Wawrzeńczyk C., Wilk K.A. (2017). Phosphatidylcholine with conjugated linoleic acid in fabrication of novel lipid nanocarriers. Colloids Surf. A Physicochem. Eng. Asp..

[158] Kerz T., Paret G., Herff H. (2007). Routes of drug administration. Cardiac Arrest, The Science and Practice of Resuscitation Medicine.

[159] Lang X., Wang T., Sun M., Chen X., Liu Y. (2020). Advances and applications of chitosan-based nanomaterials as oral delivery carriers: A review. Int. J. Biol. Macromol..

[160] Mrsny R.J. (2012). Oral drug delivery research in Europe. J. Control. Release.

[161] Kerns E.H., Di L. (2008). Drug-Like Properties: Concepts, Structure Design and Methods.

[162] Homar M., Cegnar M., Kotnik M., Peternel L. (2010). Toward effective long-term prevention of thromboembolism: Novel oral anticoagulant delivery systems. Semin. Thromb. Hemost..

[163] Acharya S.D., Tamane P.K., Khante S.N., Pokharkar V.B. (2020). QbD based optimization of curcumin nanoemulsion: DoE and cytotoxicity studies. Indian, J. Pharm. Educ. Res..

[164] Borrin T.R., Georges E.L., Moraes I.C.F., Pinho S.C. (2016). Curcumin-loaded nanoemulsions produced by the emulsion inversion point (EIP) method: An evaluation of process parameters and physico-chemical stability. J. Food Eng..

[165] Shukla M., Jaiswal S., Sharma A., Srivastava P.K., Arya A., Dwivedi A.K., Lal J. (2017). A combination of complexation and self-nanoemulsifying drug delivery system for enhancing oral bioavailability and anticancer efficacy of curcumin. Drug Dev. Ind. Pharm..

[166] Yin J., Hou Y., Song X., Wang P., Li Y. (2019). Cholate-modified polymer-lipid hybrid nanoparticles for oral delivery of quercetin to potentiate the antileukemic effect. Int. J. Nanomed..

[167] Senna J.P., Barradas T.N., Cardoso S., Castiglione T.C., Serpe M.J., Silva K.G.d.H.e., Mansur C.R.E. (2018). Dual alginate-lipid nanocarriers as oral delivery systems for amphotericin B. Colloids Surf. B Biointerfaces.

[168] Ćetković Z., Cvijić S., Vasiljević D. (2019). Formulation and characterization of novel lipid-based drug delivery systems containing polymethacrylate polymers as solid carriers for sustained release of simvastatin. J. Drug Deliv. Sci. Technol..

[169] Hädrich G., Monteiro S.O., Rodrigues M.R., De Lima V.R., Putaux J.L., Bidone J., Teixeira H.F., Muccillo-Baisch A.L., Dora C.L. (2016). Lipid-based nanocarrier for quercetin delivery: System characterization and molecular interactions studies. Drug Dev. Ind. Pharm..

[170] Jhan S., Pethe A.M. (2020). Double-loaded liposomes encapsulating lycopene β-cyclodextrin complexes: Preparation, optimization, and evaluation. J. Liposome Res..

[171] Bai C., Zheng J., Zhao L., Chen L.L., Xiong H., McClements D.J. (2019). Development of Oral Delivery Systems with Enhanced Antioxidant and Anticancer Activity: Coix Seed Oil and β-Carotene Coloaded Liposomes. J. Agric. Food Chem..

[172] Pangeni R., Panthi V.K., Yoon I.S., Park J.W. (2018). Preparation, characterization, and in vivo evaluation of an oral multiple nanoemulsive system for co-delivery of pemetrexed and quercetin. Pharmaceutics.

[173] Gupta B.K., Kumar S., Kaur H., Ali J., Baboota S. (2018). Attenuation of oxidative damage by coenzyme Q10 loaded nanoemulsion through oral route for the management of Parkinson’s disease. Rejuvenation Res..

[174] Li J., Hwang I.C., Chen X., Park H.J. (2016). Effects of chitosan coating on curcumin loaded nano-emulsion: Study on stability and in vitro digestibility. Food Hydrocoll..

[175] Vecchione R., Quagliariello V., Calabria D., Calcagno V., De Luca E., Iaffaioli R.V., Netti P.A. (2016). Curcumin bioavailability from oil in water nano-emulsions: In vitro and in vivo study on the dimensional, compositional and interactional dependence. J. Control. Release.

[176] Pangeni R., Kang S.W., Oak M., Park E.Y., Park J.W. (2017). Oral delivery of quercetin in oil-in-water nanoemulsion: In vitro characterization and in vivo anti-obesity efficacy in mice. J. Funct. Foods.

[177] Teixeira M.C., Severino P., Andreani T., Boonme P., Santini A., Silva A.M., Souto E.B. (2017). D-α-tocopherol nanoemulsions: Size properties, rheological behavior, surface tension, osmolarity and cytotoxicity. Saudi Pharm. J..

[178] Imran M., Shah M.R., Ullah F., Ullah S., Elhissi A.M.A., Nawaz W., Ahmad F., Sadiq A., Ali I. (2016). Sugar-based novel niosomal nanocarrier system for enhanced oral bioavailability of levofloxacin. Drug Deliv..

[179] Tian C., Asghar S., Wu Y., Amerigos D.K., Chen Z., Zhang M., Yin L., Huang L., Ping Q., Xiao Y. (2017). N-acetyl-l-cysteine functionalized nanostructured lipid carrier for improving oral bioavailability of curcumin: Preparation, in vitro and in vivo evaluations. Drug Deliv..

[180] Sun R., Xia Q. (2019). Nanostructured lipid carriers incorporated in alginate hydrogel: Enhanced stability and modified behavior in gastrointestinal tract. Colloids Surf. A Physicochem. Eng. Asp..

[181] Elmowafy M., Ibrahim H.M., Ahmed M.A., Shalaby K., Salama A., Hefesha H. (2017). Atorvastatin-loaded nanostructured lipid carriers (NLCs): Strategy to overcome oral delivery drawbacks. Drug Deliv..

[182] Fathi H.A., Allam A., Elsabahy M., Fetih G., El-Badry M. (2018). Nanostructured lipid carriers for improved oral delivery and prolonged antihyperlipidemic effect of simvastatin. Colloids Surf. B Biointerfaces.

[183] Chen G., Wang K., Zhou Y., Ding L., Ullah A., Hu Q., Sun M., Oupický D. (2016). Oral Nanostructured Lipid Carriers Loaded with Near-Infrared Dye for Image-Guided Photothermal Therapy. ACS Appl. Mater. Interfaces.

[184] Bazylińska U., Pucek A., Sowa M., Matczak-Jon E., Wilk K.A. (2014). Engineering of phosphatidylcholine-based solid lipid nanocarriers for flavonoids delivery. Colloids Surf. A Physicochem. Eng. Asp..

[185] Ramalingam P., Ko Y.T. (2015). Enhanced oral delivery of curcumin from N-trimethyl chitosan surface-modified solid lipid nanoparticles: Pharmacokinetic and brain distribution evaluations. Pharm. Res..

[186] Dudhipala N., Veerabrahma K. (2017). Improved anti-hyperlipidemic activity of Rosuvastatin Calcium via lipid nanoparticles: Pharmacokinetic and pharmacodynamic evaluation. Eur. J. Pharm. Biopharm..

[187] Kazi M., Al-Qarni H., Alanazi F.K. (2017). Development of oral solid self-emulsifying lipid formulations of risperidone with improved in vitro dissolution and digestion. Eur. J. Pharm. Biopharm..

[188] Khattab A., Hassanin L., Zaki N. (2017). Self-Nanoemulsifying Drug Delivery System of Coenzyme (Q10) with Improved Dissolution, Bioavailability, and Protective Efficiency on Liver Fibrosis. AAPS PharmSciTech.

[189] Alwadei M., Kazi M., Alanazi F.K. (2019). Novel oral dosage regimen based on self-nanoemulsifying drug delivery systems for codelivery of phytochemicals–Curcumin and thymoquinone. Saudi Pharm. J..

[190] Ahmad N., Ahmad R., Naqvi A.A., Alam M.A., Abdur Rub R., Ahmad F.J. (2017). Enhancement of Quercetin Oral Bioavailability by Self-Nanoemulsifying Drug Delivery System and their Quantification Through Ultra High Performance Liquid Chromatography and Mass Spectrometry in Cerebral Ischemia. Drug Res. (Stuttg)..

[191] Tripathi S., Kushwah V., Thanki K., Jain S. (2016). Triple antioxidant SNEDDS formulation with enhanced oral bioavailability: Implication of chemoprevention of breast cancer. Nanomed. Nanotechnol., Biol. Med..

[192] Kumar R., Khursheed R., Kumar R., Awasthi A., Sharma N., Khurana S., Kapoor B., Khurana N., Singh S.K., Gowthamarajan K. (2019). Self-nanoemulsifying drug delivery system of fisetin: Formulation, optimization, characterization and cytotoxicity assessment. J. Drug Deliv. Sci. Technol..

[193] Qin L., Niu Y., Wang Y., Chen X. (2018). Combination of Phospholipid Complex and Submicron Emulsion Techniques for Improving Oral Bioavailability and Therapeutic Efficacy of Water-Insoluble Drug. Mol. Pharm..

[194] Condat M., Babinot J., Tomane S., Malval J.P., Kang I.K., Spillebout F., Mazeran P.E., Lalevée J., Andalloussi S.A., Versace D.L. (2016). Development of photoactivable glycerol-based coatings containing quercetin for antibacterial applications. RSC Adv..

[195] Orozco D., Skamarack J., Reins K., Titlow B., Lunetta S., Li F., Roman M. (2007). Determination of ubidecarenone (coenzyme Q10, ubiquinol-10) in raw materials and dietary supplements by high-performance liquid chromatography with ultraviolet detection: Single-laboratory validation. J. AOAC Int..

[196] Prasad T., Kalaiselvan T., Surabhi S., Vivek D., Ranvirkumar S., Gyanendra Nath S. (2014). Atorvastatin Induced Vasculitis. Indian J. Pharm. Pract..

[197] Mikulich A.V., Tretyakova A.I., Knukshto V.N., Plavskaya L.G., Leusenka I.A., Ananich T.S., Plavskii V.Y., Ulaschik V.S. (2018). Potential of Antifungal Drugs as Photosensitizers. KnE Energy.

